# PLK1 stabilizes β-catenin to drive colorectal carcinogenesis through NFKB2-mediated transcriptional activation of USP2a and site-specific phosphorylation

**DOI:** 10.7150/thno.122368

**Published:** 2026-01-01

**Authors:** Yan Li, Lili Zhao, Jiaqiang Deng, Songmei Lu, Jingyu Du, Chen Chen, Li Zhao, Zhiyun Xu, Wencan Wang, Yundi Wang, Fangdong Zou

**Affiliations:** College of Life Sciences, Key Laboratory of Bio-Resources and Eco-Environment of Ministry of Education, Sichuan University, Chengdu, 610065, China.

**Keywords:** PLK1, colorectal cancer, β-catenin, USP2a, NFKB2, Wnt signaling

## Abstract

**Rationale:** The Wnt/β-catenin signaling pathway is crucial in driving colorectal cancer (CRC), but therapeutic targeting is difficult due to on-target toxicity and adaptive resistance. Polo-like kinase 1 (PLK1), an essential regulator of mitosis, is known to stabilize β-catenin in various cancers. However, its specific mechanistic role in CRC, especially regarding the regulation of β-catenin ubiquitination, remains unclear.

**Methods:** We integrated RNA-seq co-expression analysis with functional studies in CRC models, employing coimmunoprecipitation, ubiquitination assays, luciferase reporter systems, site-directed mutagenesis, and *in vivo* xenograft experiments.

**Results:** We identified a dual-axis mechanism through which PLK1 posttranslationally and transcriptionally controls β-catenin stability. First, PLK1 directly phosphorylates β-catenin at Ser311, which facilitates its recruitment to the deubiquitinating enzyme USP2a, thereby shielding β-catenin from proteasomal degradation. Second, PLK1 activates the transcription factor NFKB2, which in turn transcriptionally upregulates USP2a, amplifying the deubiquitination capacity of cells. This coordinated regulation ensures robust β-catenin nuclear accumulation and activation of downstream targets such as c-Myc and Cyclin D1. Inhibiting PLK1, either genetically or pharmacologically, leads to β-catenin destabilization and reduces CRC proliferation *in vitro* and *in vivo*. Rescue experiments established a mechanistic hierarchy: USP2a overexpression cannot restore β-catenin stability when Ser311 phosphorylation is abolished, whereas NFKB2 restoration rescues USP2a expression but not the PLK1 activity-dependent β-catenin‒USP2a interaction.

**Conclusion:** Our study identified PLK1 as a key regulator of β-catenin signaling flexibility in CRC, coordinating kinase-dependent and transcriptional mechanisms to sustain pathway activation. The discovery of the PLK1-NFKB2-USP2a-β-catenin axis provides a novel therapeutic rationale for targeting PLK1 to selectively disrupt Wnt-driven tumorigenesis, potentially overcoming the toxicity limitations of conventional Wnt inhibitors.

## Introduction

Colorectal cancer (CRC) is a major cause of cancer-related deaths globally, with over 80% of cases involving abnormal activation of the Wnt/β-catenin signaling pathway 1,2. The Wnt/β-catenin cascade is a highly conserved pathway critical for embryonic development and tissue homeostasis, mediating fundamental processes such as stem cell self-renewal and epithelial‒mesenchymal transition 3,4,23,24. Its molecular mechanism involves a well-orchestrated series of events upon Wnt ligand binding to Frizzled receptors: this interaction inhibits the β-catenin destruction complex, prevents β-catenin phosphorylation and ubiquitination, and promotes its cytoplasmic accumulation and subsequent nuclear translocation, where it partners with TCF/LEF transcription factors to initiate target gene expression 5,25,26. In CRC, this pathway is constitutively activated, driving uncontrolled tumor cell proliferation and representing a validated therapeutic target 6,27. Understanding the molecular mechanisms responsible for its abnormal activation in CRC is essential for creating effective treatments.

While therapeutic targeting of this pathway holds considerable promise, its clinical efficacy has been hampered by compensatory feedback mechanisms and on-target toxicity to normal stem cells 6. This has prompted a strategic shift toward identifying upstream regulators that can selectively destabilize β-catenin in malignant cells—particularly those with established druggability—to preserve physiological Wnt functions in healthy tissues.

Polo-like kinase 1 (PLK1), a master regulator of mitotic progression, has emerged as a compelling candidate. In addition to its canonical roles in centrosome maturation, chromosome segregation, and cytokinesis, PLK1 drives tumorigenesis across various malignancies. PLK1 stabilizes β-catenin via site-specific phosphorylation in both non-small cell lung cancer (NSCLC) and esophageal squamous cell carcinoma (ESCC) 7. The frequent coamplification of PLK1 and β-catenin in many cancers is strongly correlated with poor prognosis and chemoresistance [8.9]. However, whether PLK1 mechanistically engages Wnt signaling in CRC—particularly through the control of β-catenin ubiquitination—remains unresolved, especially given the tissue-specific nature of PLK1 interactomes.

The ubiquitin-proteasome system meticulously regulates β-catenin stability. While the APC/GSK3β/CK1α destruction complex mediates its phosphorylation-dependent degradation, deubiquitinating enzymes (DUBs) counteract this process to facilitate β-catenin nuclear accumulation and transcriptional activity 10,11. Among DUBs, ubiquitin-specific protease 2a (USP2a) has garnered significant attention for its oncogenic roles in stabilizing proteins such as PD-L1, Cyclin D1, and Twist across various cancers 12. Furthermore, USP2a is transcriptionally activated by NF-κB family members—a pathway itself modulated by PLK1 in other contexts 13. Despite these connections, the direct links between PLK1, NFKB2 (a key NF-κB subunit), and USP2a in CRC remain unexplored, creating a critical knowledge gap 14.

Herein, we address two interdependent mechanistic voids that impede therapeutic progress. First, while PLK1 is known to phosphorylate β-catenin at Ser60 to regulate mitotic fidelity 15, whether this or other phosphorylation events influence USP2a-mediated deubiquitination in the unique molecular landscape of CRC is unknown. Second, although PLK1 can stabilize the β-catenin protein, its capacity to coopt the NFKB2 transcription factor for transcriptional control of USP2a remains entirely uncharted 16,17. Resolving these questions is clinically urgent, as early-phase trials of PLK1 inhibitors (e.g., onvansertib) show promise in patients with KRAS-mutant CRC, yet predictive biomarkers for patient stratification are critically lacking 18.

In this study, we establish that PLK1 stabilizes β-catenin through a convergent dual mechanism in colorectal cancer: it transcriptionally induces USP2a via NFKB2 activation while simultaneously phosphorylating β-catenin at Ser311 to prime its recruitment to USP2a. By orchestrating this sophisticated regulatory circuit, PLK1 functionally links the mitotic machinery to Wnt pathway addiction, revealing a therapeutically targetable vulnerability that may circumvent the toxicity limitations of conventional Wnt inhibitors.

## Results

### PLK1 is aberrantly overexpressed in CRC and drives malignant progression

**Figure 1:**
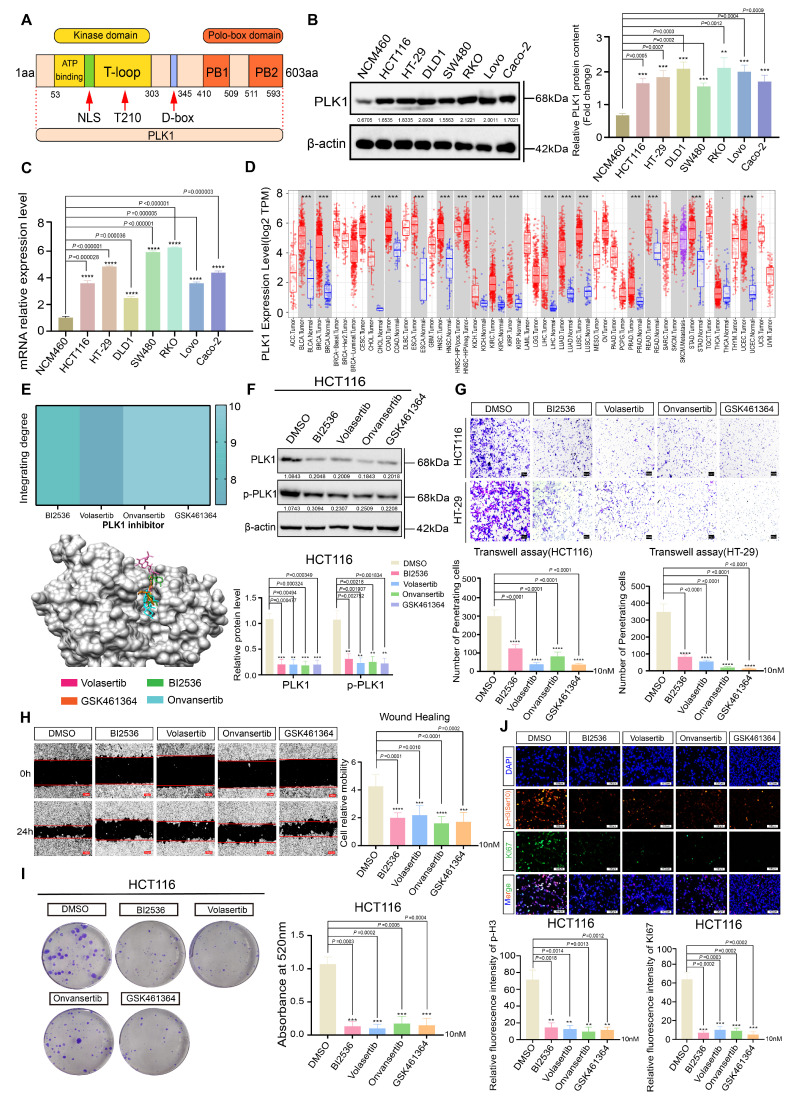
** PLK1 is aberrantly overexpressed in CRC and drives malignant phenotypes. (A)** Schematic representation of the PLK1 protein structure, highlighting the N-terminal kinase domain (KD) containing the ATP-binding pocket, nuclear localization signal (NLS), and Thr-loop phosphorylation site, and the C-terminal polo-box domain (PBD).** (B)** Western blot analysis of PLK1 protein expression in CRC cell lines versus NCM460 cells. β-Actin served as a loading control. Blots are representative of three independent experiments. **(C)** PLK1 mRNA expression levels in seven CRC cell lines compared with those in the normal intestinal epithelial cell line NCM460, as determined by quantitative RT-PCR. The data are presented as the means ± SDs (n = 3). **(D)** Analysis of PLK1 gene expression across 17 human cancer types from the TIMER database, comparing tumor tissues with adjacent normal tissues (where available). Box plots displaying the median, interquartile range, and outliers (n ≥ 50 per group). **(E)** Molecular docking models and binding scores illustrating the interaction of PLK1 inhibitors (BI2536, volasertib, onvansertib, and GSK461364) with the ATP-binding pocket of the PLK1 kinase domain. **(F)** Western blot analysis of PLK1 and phospho-PLK1 (Thr210) protein levels in HCT116 cells following 24 hours of treatment with a 10 nM PLK1 inhibitor. β-Actin was used as a loading control. **(G)** Cell invasion of HCT116 and HT-29 cells after 16 hours of pretreatment with a 10 nM PLK1 inhibitor was assessed via a Transwell assay. Invaded cells were stained and quantified after 24 hours. Representative images are shown (scale bar: 100 μm). **(H)** Cell migration was evaluated via a scratch wound healing assay in HCT116 cells pretreated with a 10 nM PLK1 inhibitor for 16 hours. Wound closure was measured at 0 and 24 hours. Representative images and quantification are shown (scale bar: 200 μm). **(I)** Colony formation capacity of HCT116 cells after 16 hours of pretreatment with a 10 nM PLK1 inhibitor, followed by 14 days of culture in standard medium. Colonies were stained and counted. The data are presented as the means ± SDs (n = 3). **(J)** Immunofluorescence staining and quantification of the proliferation markers KI67 and phospho-histone H3 (Ser10) in HCT116 cells treated with a 10 nM PLK1 inhibitor for 24 hours. The data represent the mean fluorescence intensity ± SD (n = 3; scale bar: 50 μm).

Polo-like kinase 1 (PLK1) is a crucial serine/threonine kinase in eukaryotic cells that regulates various phases of cell division, such as spindle assembly, centrosome maturation, and cytokinesis 13, 19. Structurally, PLK1 features an N-terminal kinase domain (KD) responsible for catalytic activity and a C-terminal polo-box domain (PBD) that mediates subcellular localization and substrate recognition (Figure 1A). Its frequent overexpression and prognostic significance across human cancers prompted us to investigate its role in colorectal cancer (CRC) 22.

Initially, we evaluated PLK1 expression in seven different CRC cell lines. Quantitative analysis revealed a significant upregulation of PLK1 at both mRNA and protein levels in comparison to normal intestinal epithelial NCM460 cells (Figure 1B, C). Interference with public databases corroborated this finding: analysis of the TIMER database revealed PLK1 overexpression in the tumor tissues of 15 out of 17 cancer types, an observation that was consistent with the combined TCGA and GTEx dataset analysis (Figure 1D). Furthermore, PLK1 transcript levels were markedly elevated in 5-fluorouracil (5FU)-resistant HCT116 sublines (FURA and FURB) compared with parental cells (Figure S1A), and increased PLK1 transcriptional activity was confirmed in mouse models of colorectal inflammation and cancer (Figure S1B), collectively linking PLK1 expression to CRC pathogenesis and therapy resistance.

To establish PLK1 as a viable therapeutic target, we performed molecular docking, which confirmed the high-affinity binding of four distinct PLK1 inhibitors (BI2536, volasertib, onvansertib, and GSK461364) to its kinase domain (Figure 1E). The reliability of our experimental system was validated by Western blotting, which revealed reduced PLK1 in inhibitor-treated and PLK1-knockdown cells (sh1-PLK1, sh2-PLK1) and elevated PLK1 in PLK1-overexpressing cells (pLenti-PLK1) (Figure 1F, S1C, D).

Functional interrogation revealed that PLK1 is a critical driver of CRC malignancy. Its inhibition significantly attenuated cell proliferation across four CRC cell lines (Figure S1E-F), impaired migratory and invasive capacities in Transwell assays and wound healing (Figure 1G, H), and reduced clonogenic potential (Figure 1I, S1I, J). Immunofluorescence analysis provided direct evidence that the intensities of the proliferation markers KI67 and phospho-histone H3 (Ser10) were diminished upon PLK1 inhibition and increased upon PLK1 overexpression (Figure 1J, S1K, L).

In conclusion, our data establish PLK1 as a commonly overexpressed and functionally critical oncokinase in CRC. Its elevation promotes aggressive cellular phenotypes, including enhanced proliferation, migration, invasion, and therapy resistance, suggesting that it is a compelling therapeutic target for further investigation.

### PLK1 promotes activation of the Wnt/β-catenin signaling pathway

**Figure 2:**
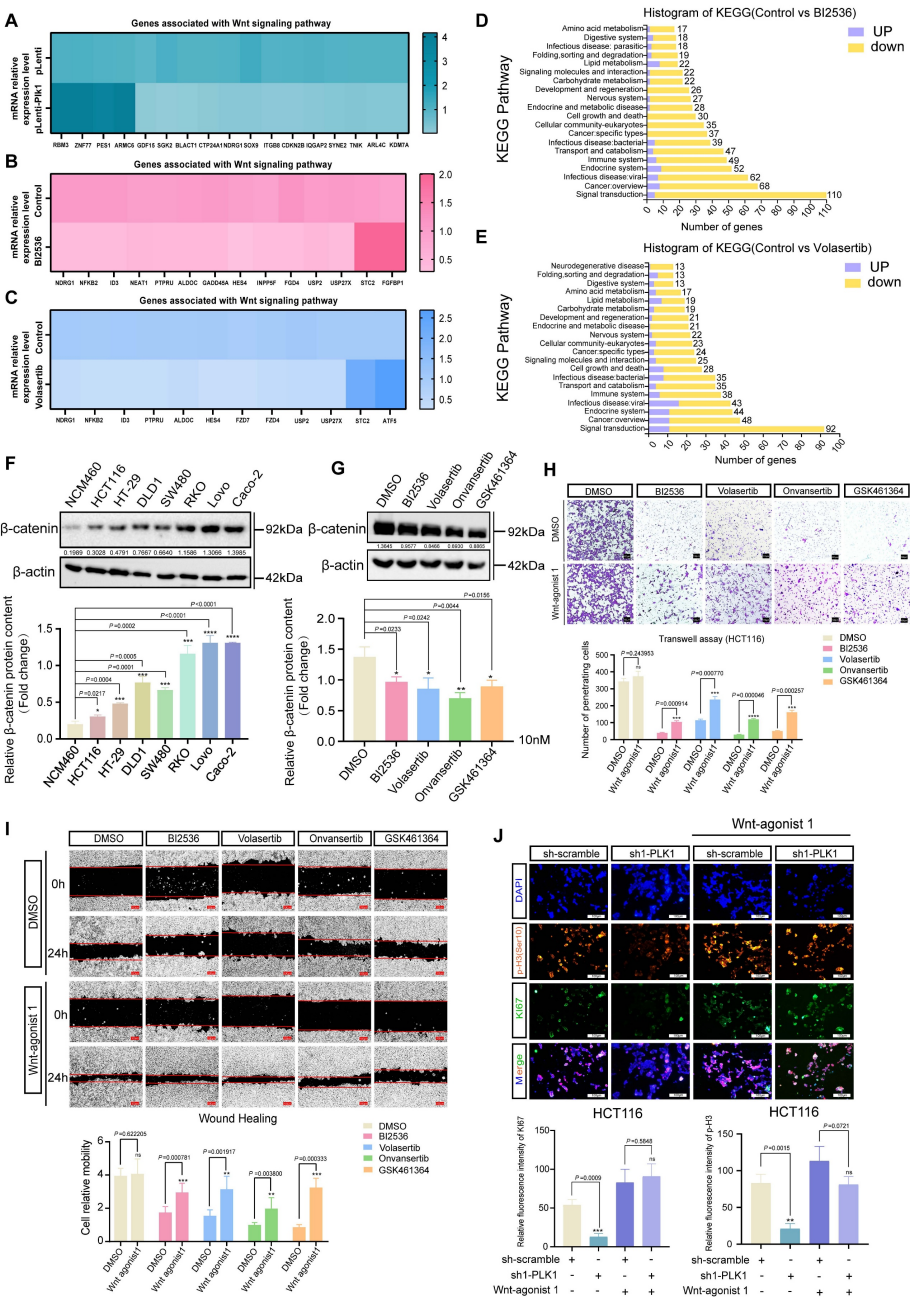
** PLK1 activates the Wnt/β-catenin signaling pathway in colorectal cancer. (A)** Gene set enrichment analysis (GSEA) of RNA-seq data from PLK1-overexpressing HCT116 cells (HCT116-pLenti-PLK1) revealed significant enrichment of Wnt/β-catenin pathway genes (NES, FDR < 0.05). **(B)** GSEA revealed downregulation of Wnt/β-catenin pathway genes in HCT116 cells treated with 1 nM BI2536 for 12 hours (NES, FDR < 0.05). **(C)** GSEA demonstrated suppression of Wnt/β-catenin signaling in HCT116 cells treated with 1 nM volasertib for 12 hours (NES, FDR < 0.05). **(D-E)** KEGG pathway enrichment analysis of BI2536 and volasertib -treated HCT116 cells revealed that Wnt/β-catenin signaling was the significantly altered pathway (*p* < 0.01). **(F)** Western blot analysis of β-catenin protein levels in CRC cell lines compared with those in normal NCM460 cells. β-Actin served as a loading control. The lower panel shows densitometric quantification (mean ± SD, n = 3; *p < 0.05, Student's t test). **(G)** β-catenin protein levels in HCT116 cells following 24 hours of treatment with 10 nM PLK1 inhibitor. The lower panel shows the results of the quantitative analysis (± SD, n = 3; *p < 0.05; representative blot shown). **(H)** Transwell invasion assay in HCT116 cells pretreated with 10 nM PLK1 inhibitor followed by 2 nM Wnt agonist. Invasion capacity was rescued by Wnt activation. Quantitative analysis shows the mean ± SD, n = 3; *p < 0.05 vs the inhibitor-only group (scale bar: 100 μm). **(I)** Scratch wound healing assay in HCT116 cells treated with the PLK1 inhibitor or the Wnt agonist. Wnt activation reversed the migration defect induced by PLK1 inhibition. The data represent the means ± SDs, n = 3; *p < 0.05 vs the inhibitor-only group (scale bar: 200 μm). **(J)** Immunofluorescence staining of the proliferation markers KI67 and p-H3 (Ser10) in PLK1-knockdown HCT116 cells treated with 2 nM Wnt agonist. Wnt activation restored proliferative signals. The data represent the mean fluorescence intensity ± SD, n = 3; *p < 0.05 vs the control (scale bar: 50 μm).

To delineate the mechanistic basis of PLK1-driven colorectal cancer (CRC) progression, we performed transcriptome sequencing on CRC cells with either PLK1 overexpression or inhibitor treatment (BI2536, Volasertib). Our analysis identified notable changes in the expression of several genes linked to the Wnt signaling pathway (Figure 2A-E), indicating that PLK1 might influence CRC malignancy by modulating this pathway. KEGG enrichment analysis further confirmed the specific enrichment of Wnt-related genes upon PLK1 perturbation (Figure S2A, B). This potential synergy between PLK1-mediated mitotic control and Wnt-driven proliferation underscores a plausible mechanism for the aggressive phenotype of CRC.

Initially, we evaluated the significance of the Wnt/β-catenin pathway within a wider oncogenic framework. Analysis of the TIMER database revealed frequent overexpression of β-catenin, the central effector of Wnt signaling, across multiple cancer types (Figure S2C). Consistent with these findings, we found that the expression of Wnt3a, an upstream activator of the pathway, was transcriptionally upregulated in all seven tested CRC cell lines compared with that in normal colonic epithelial NCM460 cells (Figure 2F). qPCR further confirmed elevated β-catenin protein levels in CRC cells (Figure S2D).

We next directly investigated the functional link between PLK1 and the Wnt pathway. The inhibition of PLK1 in HCT116 cells led to the transcriptional downregulation of key Wnt target genes, including FZD1, FZD3, FZD4, FZD7, LGR4, NKD1, and USP2, whereas PLK1 overexpression had the opposite effect (Figure S2E, F). At the protein level, PLK1 inhibition reduced, while PLK1 overexpression increased, β-catenin abundance (Figure 2G, S2G), establishing PLK1 as a positive regulator of β-catenin in CRC.

To determine whether the oncogenic functions of PLK1 are mediated through Wnt pathway activation, we performed rescue experiments using a Wnt agonist (Wnt agonist 1) in PLK1-inhibited CRC cells (RKO, HCT116, DLD1, and HT-29). Remarkably, Wnt activation substantially reversed the phenotypic deficits induced by PLK1 inhibition: colony formation recovered to 83% of the control levels (Figure S2H); cell invasion and migration were restored by 71% and 65%, respectively (Figure 2H, I); cell proliferation, as assessed by the CCK-8 assay, was rescued by 68-92% (Figure S2I-L); and the fluorescence intensities of the proliferation markers KI67 and p-H3 (Ser10) returned to 89-95% of the baseline values (Figure 2J, S2M). These data demonstrate that Wnt pathway activation is a major downstream effector of PLK1-driven malignancy.

Furthermore, to unequivocally confirm that the observed effects of small-molecule inhibitors are due to on-target kinase inhibition rather than off-target toxicity, we employed critical genetic control. We constructed a kinase-dead PLK1 mutant (PLK1-K82R), which specifically disrupts binding to ATP-competitive inhibitors such as BI2536 while preserving basal protein expression. In cells reconstituted with wild-type PLK1 (PLK1-WT), BI2536 treatment reduced the levels of PLK1, phospho-PLK1 (T210), and β-catenin. In contrast, in cells expressing the PLK1-K82R mutant, BI2536 failed to bind PLK1 and consequently did not reduce PLK1 protein levels or affect β-catenin stability (Figure S2N, O). This genetic evidence confirms that the downregulation of total PLK1 protein expression by inhibitors is a secondary effect potentially linked to cell cycle arrest and subsequent degradation, whereas the core mechanism of β-catenin regulation is unequivocally dependent on PLK1 kinase activity.

Our study demonstrates that PLK1 facilitates the malignant progression of CRC by sustaining the activation of the Wnt/β-catenin signaling pathway. This kinase-dependent function operates in parallel with its canonical role in mitotic regulation, providing a comprehensive mechanistic basis for its oncogenic activity.

### PLK1 promotes the stability of β-catenin by regulating its ubiquitination

**Figure 3:**
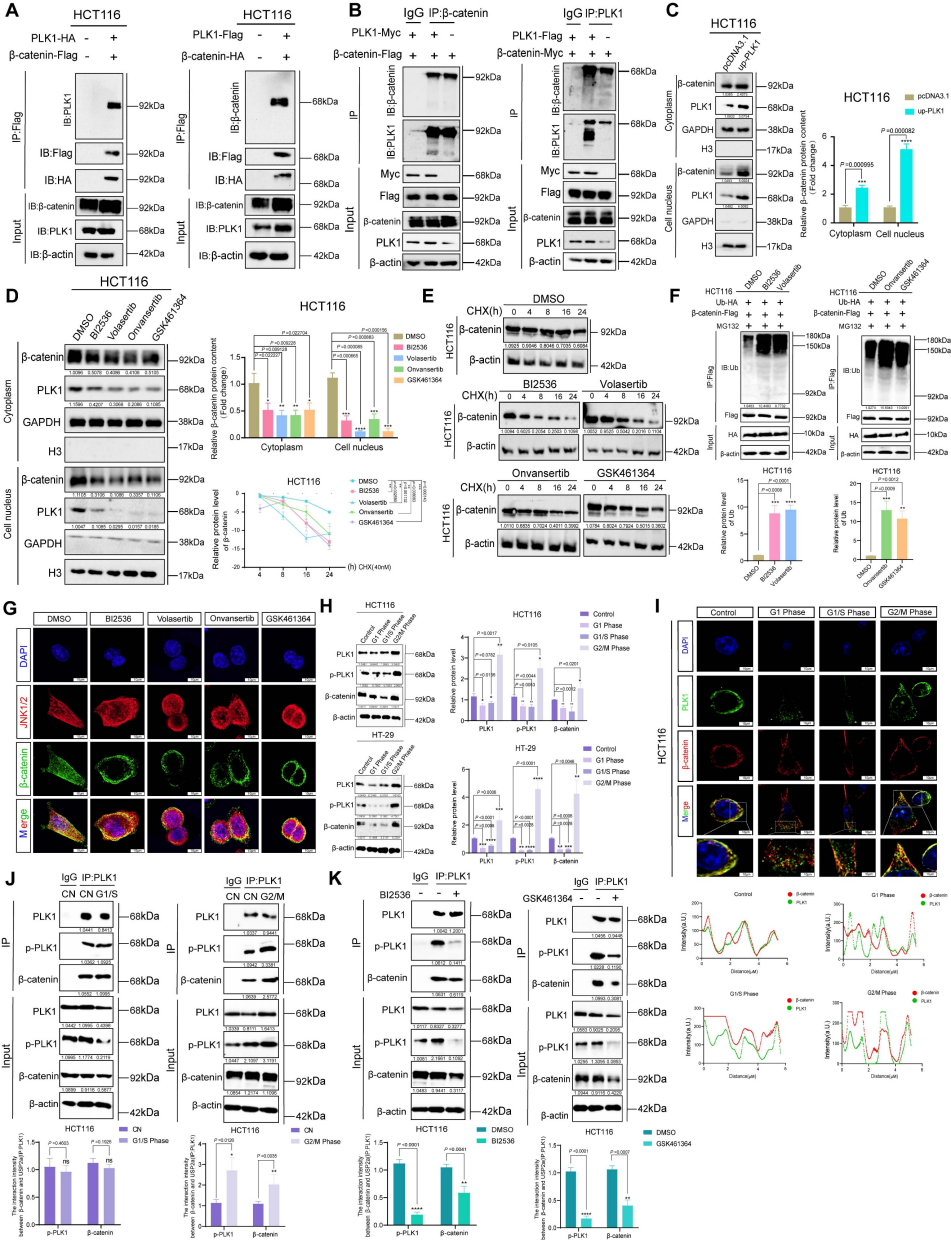
** PLK1 stabilizes β-catenin by suppressing its ubiquitin-mediated degradation. (A)** Co-immunoprecipitation (Co-IP) assays demonstrated a direct interaction between PLK1 and β-catenin. HCT116 cells were cotransfected with β-catenin-Flag and PLK1-HA for 48 h, followed by immunoprecipitation with anti-Flag beads and detection of PLK1-HA. Reciprocal Co-IP using PLK1-Flag and β-catenin-HA confirmed bidirectional binding.** (B)** Endogenous Co-IP results validating physiological interactions. HCT116 cells overexpressing PLK1-Myc and β-catenin-Flag were immunoprecipitated with an anti-β-catenin antibody, which revealed that PLK1-Myc coprecipitated. Reverse Co-IP with an anti-PLK1 antibody revealed β-catenin-Flag enrichment.** (C)** Nucleocytoplasmic fractionation showing that PLK1 overexpression increases β-catenin levels in both the nuclear and cytoplasmic fractions of HCT116 cells.** (D)** PLK1 inhibition (10 nM, 24 h) reduces β-catenin accumulation in both the nuclear and the cytoplasmic compartments.** (E)** Protein stability assay using cycloheximide (CHX, 40 nM) chase. PLK1 inhibition significantly shortens the β-catenin half-life in HCT116 cells. **(F)** Ubiquitination assays revealed that PLK1 inhibition increases β-catenin ubiquitination. HCT116 cells co-expressing β-catenin-Flag and Ub-HA were treated with MG132 (10 nM, 48 h) and a PLK1 inhibitor (10 nM, 24 h). **(G)** Confocal microscopy (SpinSR) revealed reduced β-catenin fluorescence intensity upon PLK1 inhibition (10 nM, 24 h). Scale bar: 10 μm. **(H)** Cell cycle synchronization via double thymidine (TdR) blockade reveals cell cycle-dependent expression patterns of PLK1, p-PLK1, and β-catenin in HCT116 and HT-29 cells. **(I)** Confocal analysis of PLK1 and β-catenin colocalization across different cell cycle phases (G1, G1/S, and G2/M) in synchronized HCT116 cells. Scale bar: 10 μm. **(J)** Endogenous Co-IP demonstrated persistent PLK1-β-catenin interactions throughout the cell cycle in synchronized HCT116 cells. **(K)** PLK1 inhibition disrupts the endogenous PLK1-β-catenin interaction. HCT116 cells treated with BI2536 or GSK461364 (10 nM, 24 h) presented reduced complex formation in Co-IP assays.

Previous studies suggest that PLK1 phosphorylates β-catenin at specific sites, influencing its function based on context. Phosphorylation at Ser311 increases β-catenin's stability and transcriptional activity in non-small cell lung cancer, while phosphorylation at Ser60 is involved in cytokinesis regulation 7, 28, 29. To elucidate the mechanism by which PLK1 regulates β-catenin in CRC, we first confirmed their direct physical interaction. Co-immunoprecipitation (co-IP) assays in HCT116 cells demonstrated that PLK1-Flag specifically coprecipitated with β-catenin-HA, and reciprocally, β-catenin-Flag pulled down PLK1-HA (Figure 3A). Endogenous co-IP further validated this mutual enrichment (Figure 3B).

Considering the crucial role of β-catenin nuclear translocation in Wnt pathway activation 30, we examined the potential influence of PLK1 on this process. Nucleocytoplasmic fractionation revealed that PLK1 overexpression increased nuclear β-catenin levels by 2.4-fold, whereas PLK1 inhibition reduced its nuclear accumulation to 45% of the control level in HCT116 cells (Figure 3C, D). This finding was corroborated by confocal imaging (Figure 3G, S3F).

We next explored the mechanistic link between PLK1 and β-catenin stability. In unstimulated cells, β-catenin is predominantly regulated by ubiquitination and subsequent proteasomal degradation 31. Pulse-chase experiments using cycloheximide (CHX) revealed that pharmacological inhibition of PLK1 significantly shortened the half-life of β-catenin in HCT116 and HT-29 cells (Figure 3E, S3A). Conversely, PLK1 overexpression increased β-catenin stability (Figure S3B). Consistent with these findings, ubiquitination assays demonstrated that PLK1 inhibition increased, whereas PLK1 overexpression suppressed, β-catenin ubiquitination (Figure 3F, S3D, S3E, S3G).

To unequivocally attribute these effects to on-target kinase activity rather than off-target inhibitor effects, we employed a kinase-dead PLK1 mutant (PLK1-K82R). Unlike wild-type PLK1 (PLK1-WT), the PLK1-K82R mutant did not enhance β-catenin stability or decrease its ubiquitination level (Figure S3B-E). This genetic evidence confirms that the regulation of β-catenin is specifically dependent on the catalytic activity of PLK1.

As PLK1 inhibition often induces multinucleation—a known consequence of mitotic failure—we sought to determine whether β-catenin regulation is secondary to this ploidy change or directly linked to PLK1 kinase activity. Synchronization of HCT116 cells via a double thymidine (TdR) block revealed that β-catenin protein levels oscillated in a cell cycle-dependent manner, peaking at the G2/M phase in parallel with PLK1 expression (Figure 3H). Colocalization and endogenous co-IP analyses across different cell cycle stages confirmed that the PLK1-β-catenin interaction persists throughout the cell cycle, with its strength correlating with overall protein levels (Figure 3I, J). However, inhibiting PLK1 kinase activity significantly weakened this interaction, even in polyploid cells (Figure 3K). These findings demonstrate that the interaction is governed by PLK1 kinase activity itself, not merely by the cell cycle stage or ploidy. The multinucleated phenotype is thus a concomitant event that does not confound the core mechanistic relationship.

In conclusion, our data establish that PLK1 binds to and stabilizes β-catenin in CRC by inhibiting its ubiquitin-mediated degradation. This mechanism is strictly dependent on PLK1 kinase activity, promotes β-catenin nuclear accumulation, and is independent of cell cycle-induced ploidy changes, confirming the role of PLK1 as a direct regulator of Wnt signaling in colorectal carcinogenesis.

### PLK1 reduces the ubiquitination of β-catenin by upregulating USP2a

**Figure 4:**
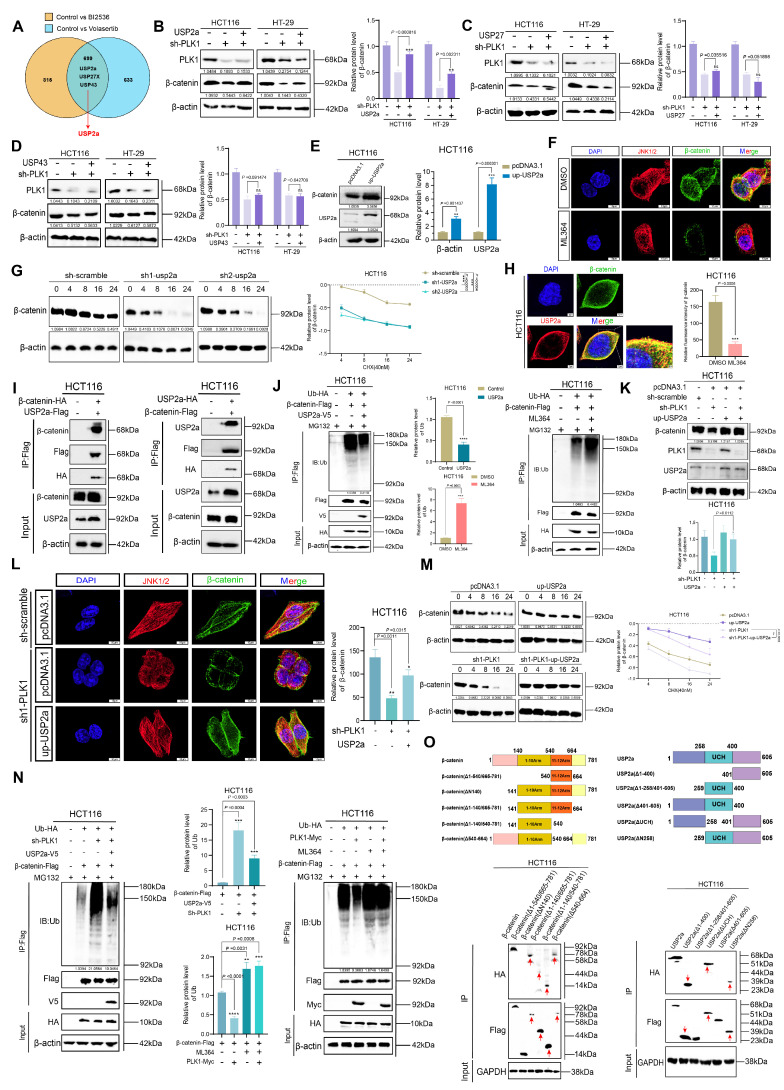
** PLK1 attenuates β-catenin ubiquitination by upregulating USP2a. (A)** Venn diagram showing that deubiquitinating enzymes (DUBs) are commonly downregulated in HCT116 cells treated with BI2536 or volasertib. **(B-D)** Functional validation of candidate DUBs in PLK1-knockdown cells: **(B)** USP2a overexpression rescues β-catenin protein levels in PLK1-knockdown HCT116/HT-29 cells. **(C)** USP27 overexpression fails to restore β-catenin expression. **(D)** USP43 overexpression had no rescue effect. **(E)** USP2a overexpression increases β-catenin protein levels in HCT116 cells. **(F)** Confocal microscopy (SpinSR) revealed reduced β-catenin fluorescence intensity following ML364 (10 nM, 24 h) treatment. Scale bar: 10 μm. **(G)** A protein stability assay revealed a shortened β-catenin half-life upon USP2a knockdown in HCT116 cells treated with CHX (40 nM). **(H)** Confocal analysis demonstrated the cytoplasmic colocalization of USP2a and β-catenin in HCT116 cells. Scale bar: 5 μm. **(I)** Co-immunoprecipitation confirmed the direct interaction between USP2a and β-catenin. Reciprocal Co-IP shows bidirectional binding in HCT116 cells co-expressing the tagged constructs. **(J)** Ubiquitination assays demonstrated the USP2a-mediated regulation of β-catenin stability. USP2a overexpression reduces β-catenin ubiquitination, whereas ML364 treatment (10 nM, 24 h) increases ubiquitination. MG132 (10 nM) was used to inhibit proteasomal degradation. **(K)** USP2a overexpression restored β-catenin protein levels in PLK1-knockdown HCT116 cells. **(L)** Confocal microscopy confirmed the recovery of β-catenin fluorescence upon USP2a overexpression in PLK1-knockdown cells. Scale bar: 10 μm. **(M)** USP2a overexpression rescues β-catenin stability in PLK1-knockdown HCT116 cells, as shown by the CHX chase assay. **(N)** USP2a overexpression reversed PLK1 knockdown-induced β-catenin ubiquitination, whereas PLK1 overexpression did not compensate for USP2a inhibition-mediated β-catenin ubiquitination. **(O)** Domain mapping defines the USP2a-β-catenin interaction interface. Co-IP assays with truncated mutants identified β-catenin Armadillo repeats 1-10 and the USP2a C-terminal domain as essential for binding.

Having established that PLK1 stabilizes β-catenin by suppressing its ubiquitination, we sought to identify the specific deubiquitinating enzyme (DUB) responsible for this process. Integration of RNA-seq data from PLK1-modulated cells and screening of Wnt pathway-associated DUBs revealed three consistently altered candidates: USP2a, USP27X, and USP43 (Figure 4A). Functional rescue experiments in PLK1-knockdown HCT116 and HT-29 cells demonstrated that only overexpression of USP2a, an isoform of USP2 31, 32, restored the downregulated β-catenin protein levels, whereas USP27X and USP43 had no significant effect (Figure 4B-D). This finding pinpointed USP2a as the key downstream effector.

We next validated the role of USP2a in regulating β-catenin. Ectopic expression of USP2a increased β-catenin protein levels, while USP2a knockdown reduced them (Figure 4E, S4A). Consistently, treatment with the USP2a inhibitor ML364 (10 nM) decreased, whereas USP2a overexpression increased, β-catenin fluorescence intensity (Figure 4F, S4B). Pulse-chase assays demonstrated that USP2a overexpression extended the half-life of β-catenin, whereas its knockdown reduced it (Figure 4G, S4C, S4D).

The direct interaction between USP2a and β-catenin in CRC cells was subsequently confirmed. Confocal microscopy revealed their colocalization (Figure 4H), and reciprocal co-IP assays demonstrated a direct physical interaction (Figure 4I). Notably, PLK1 did not directly bind USP2a (Figure S4E), indicating an indirect regulatory relationship. Critically, ubiquitination assays confirmed the functional consequences of this interaction: USP2a overexpression reduced, whereas ML364 treatment increased, β-catenin ubiquitination levels (Figure 4J).

We conducted a series of rescue experiments to confirm USP2a as the key mediator of PLK1. The overexpression of USP2a in PLK1-knockdown cells rescued β-catenin protein levels (Figure 4K) and fluorescence intensity (Figure 4L), restored its stability (Figure 4M), and normalized its ubiquitination levels (Figure 4N). Conversely, inhibition of USP2a with ML364 abrogated the ability of PLK1 overexpression to stabilize β-catenin (Figure 4N). These data conclusively demonstrate that PLK1 regulates β-catenin stability in a USP2a-dependent manner.

We subsequently performed subcellular fractionation followed by co-IP, which confirmed that the USP2a-β-catenin interaction occurs predominantly in the cytoplasm and is weakened upon PLK1 inhibition (Figure S4F, G). Furthermore, we investigated the seemingly paradoxical observation that USP2a overexpression could partially rescue β-catenin ubiquitination in PLK1-deficient cells. A dose-dependent co-IP experiment revealed that increasing concentrations of USP2a enhance its binding to β-catenin (Figure S4H), suggesting a "mass action effect" that can partially compensate for the lack of PLK1-mediated priming.

Finally, to map the interaction interface, we systematically generated a series of truncation mutants for both β-catenin and USP2a. Co-IP experiments revealed that β-catenin requires an intact 1-10 Armadillo repeat domain (which encompasses Ser311) for binding, whereas USP2a requires its C-terminal domain (Figure 4O, S4I). This structural insight solidifies our mechanistic model and provides a molecular basis for phospho-primed regulation.

In summary, we identified USP2a as the crucial DUB through which PLK1 stabilizes β-catenin. PLK1-mediated signaling promotes the transcription of USP2a, which in turn directly binds to and deubiquitinates β-catenin in the cytoplasm, ultimately leading to its nuclear accumulation and transcriptional activation.

### Activation of NFKB2 by PLK1 promotes the transcriptional activity of USP2a

**Figure 5:**
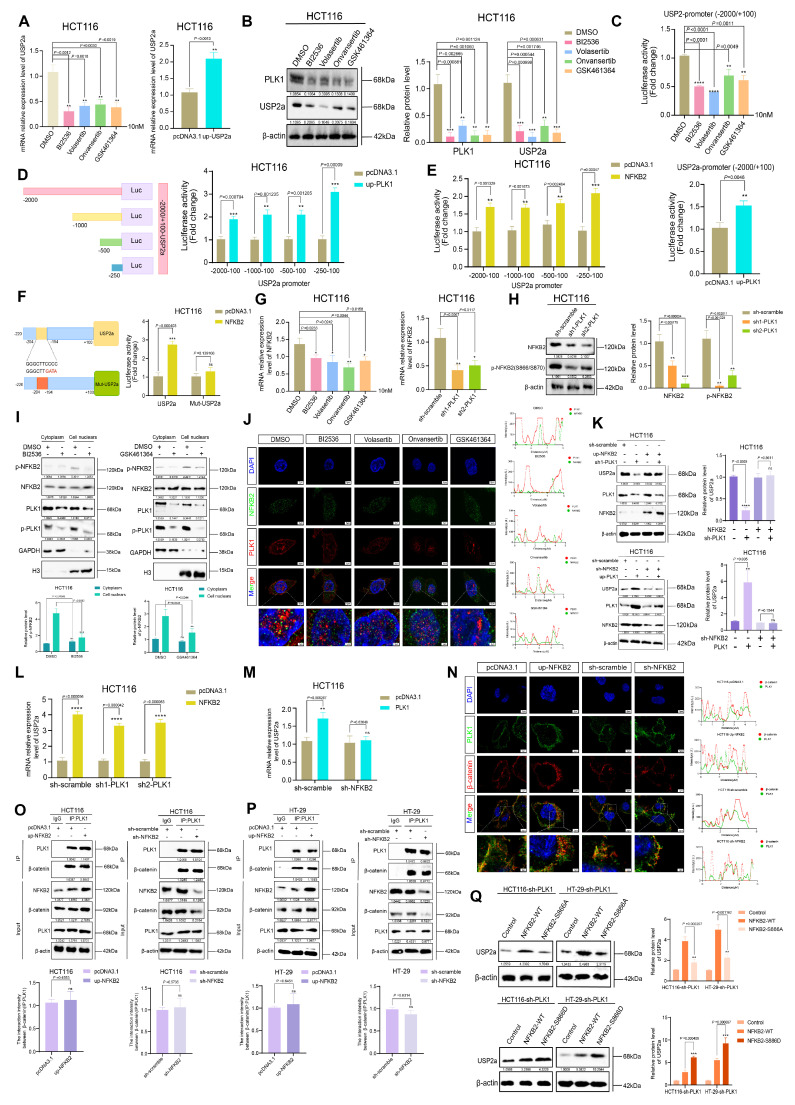
** PLK1 activates NFKB2 to promote USP2a transcription. (A)** qPCR analysis revealed that PLK1 inhibition (10 nM, 24 h) decreased, while PLK1 overexpression increased, USP2a mRNA levels in HCT116 cells. **(B)** Western blot analysis revealed reduced USP2a protein levels following PLK1 inhibition (10 nM, 48 h) in HCT116 cells. **(C)** Dual-luciferase reporter assays revealed that PLK1 inhibition suppresses, while PLK1 overexpression enhances, USP2a promoter activity in HCT116 cells. **(D)** Truncation analysis of the USP2a promoter revealed that the -250/+100 region is responsive to PLK1 regulation in HCT116 cells. **(E)** NFKB2 overexpression specifically activated the USP2a -250/+100 promoter region in luciferase assays. **(F)** Mutation of the NFKB2 binding site (Mut-USP2a) abrogates PLK1-mediated promoter activation in HCT116 cells. **(G)** qPCR analysis revealed that PLK1 inhibition or knockdown reduces NFKB2 mRNA levels in HCT116 cells. **(H)** PLK1 knockdown decreases phospho-NFKB2 protein levels in HCT116 cells. **(I)** Subcellular fractionation showing that PLK1 inhibition reduces nuclear phospho-NFKB2 levels in HCT116 cells. **(J)** Confocal microscopy (SpinSR) demonstrated that PLK1 inhibition disrupted PLK1-NFKB2 nuclear colocalization in HCT116 cells. Scale bar: 5 μm.** (K)** NFKB2 overexpression rescues USP2a protein levels in PLK1-knockdown HCT116 cells. PLK1 overexpression fails to restore USP2a expression in NFKB2-knockdown HCT116 cells. **(L)** NFKB2 overexpression rescues USP2a mRNA levels in PLK1-knockdown HCT116 cells.** (M)** PLK1 overexpression did not restore USP2a mRNA levels in NFKB2-knockdown HCT116 cells. **(N)** NFKB2 modulation (overexpression or knockdown) does not affect PLK1-β-catenin colocalization in HCT116 cells. Scale bar: 5 μm. **(O)** Endogenous Co-IP confirmed that NFKB2 expression does not affect the PLK1-β-catenin interaction in HCT116 cells. **(P)** Reciprocal Co-IP in HT-29 cells confirmed that NFKB2 does not compete with β-catenin for PLK1 binding. **(Q)** Reconstitution with phospho-mimetic NFKB2 (NFKB2-S866D), but not phospho-dead NFKB2 (NFKB2-S866A), rescues USP2a expression in PLK1-knockdown HCT116 and HT-29 cells.

Having demonstrated that PLK1 regulates β-catenin stability via USP2a, we next investigated the mechanism underlying PLK1-mediated control of USP2a expression. qPCR analysis revealed that PLK1 inhibition decreased, whereas PLK1 overexpression increased, USP2a mRNA levels (Figure 5A). Consistent with these findings, Western blotting revealed that PLK1 inhibition or knockdown reduced USP2a protein levels, whereas PLK1 overexpression upregulated them (Figure 5B, S5A, S5B), suggesting transcriptional regulation.

Analysis of our PLK1 RNA-seq data indicated a positive correlation between transcript levels of PLK1 and USP2a. To test whether PLK1 directly regulates USP2a promoter activity, we performed dual-luciferase reporter assays using the USP2a promoter region (-2000/+100). PLK1 inhibition suppressed but PLK1 overexpression increased promoter activity (Figure 5C). Truncation analysis identified the -250/+100 region of the USP2a promoter as the key segment responsive to PLK1 regulation (Figure 5D).

Bioinformatic screening of potential transcription factors that bind to this promoter region via the UCSC, String, and GeneMANIA databases identified NFKB2 as a top candidate. NFKB2, a member of the NF-κB family featuring a DNA-binding domain and ankyrin repeats (Figure S5C), was subsequently validated functionally. Luciferase assays confirmed that NFKB2 overexpression specifically activated the USP2a -250/+100 promoter region (Figure 5E). JASPAR database analysis predicted a high-affinity NFKB2 binding site at USP2a -204/-194. Mutation of this site (Mut-USP2a) completely abrogated NFKB2-mediated promoter activation (Figure 5F), confirming its necessity.

We then examined the PLK1-NFKB2 relationship. PLK1 inhibition or knockdown reduced NFKB2 mRNA levels (Figure 5G) and decreased the protein levels of phosphorylated NFKB2 (Ser866/870) (Figure 5H, S5D). This phosphorylation is critical for NFKB2 transcriptional activity. To delineate this regulation spatially, we performed subcellular fractionation, which revealed that PLK1 inhibition specifically reduced nuclear phospho-NFKB2 levels (Figure 5I). Confocal microscopy confirmed the nuclear colocalization of PLK1 and NFKB2, which was diminished upon PLK1 inhibition and enhanced by PLK1 overexpression (Figure 5J, S5E). Rescue experiments confirmed the epistatic relationship: NFKB2 overexpression rescued USP2a downregulation in PLK1-deficient cells, whereas PLK1 overexpression failed to restore USP2a expression upon NFKB2 knockdown (Figure 5K-M).

Finally, to confirm the independence of the two proposed arms of the PLK1 regulatory network, we tested whether NFKB2 competes with β-catenin for PLK1 binding. Endogenous co-IP and colocalization analyses in HCT116 and HT-29 cells revealed that changes in NFKB2 expression had no impact on the PLK1-β-catenin interaction (Figure 5N, O, P). These findings indicate that the PLK1-NFKB2-USP2a transcriptional axis and the PLK1-β-catenin phosphorylation/deubiquitination axis represent spatially and functionally distinct pathways. Most definitively, reconstitution of PLK1-knockdown cells with phospho-mimetic NFKB2 (NFKB2-S866D) more effectively restored USP2a levels than did reconstitution with wild-type NFKB2, while a phospho-dead mutant (NFKB2-S866A) was ineffective (Figure 5Q). These findings establish that PLK1-mediated phosphorylation is crucial for NFKB2 transactivation of USP2a.

In conclusion, we delineated a coherent signaling cascade in which PLK1 activates the transcription factor NFKB2, which in turn binds to and transactivates the USP2a promoter. This PLK1-NFKB2-USP2a axis operates independently of, and in parallel to, the direct posttranslational regulation of β-catenin by PLK1, constituting a dual-mechanistic framework for PLK1-driven oncogenesis.

### PLK1 phosphorylation of β-catenin promotes its recruitment to USP2a

**Figure 6:**
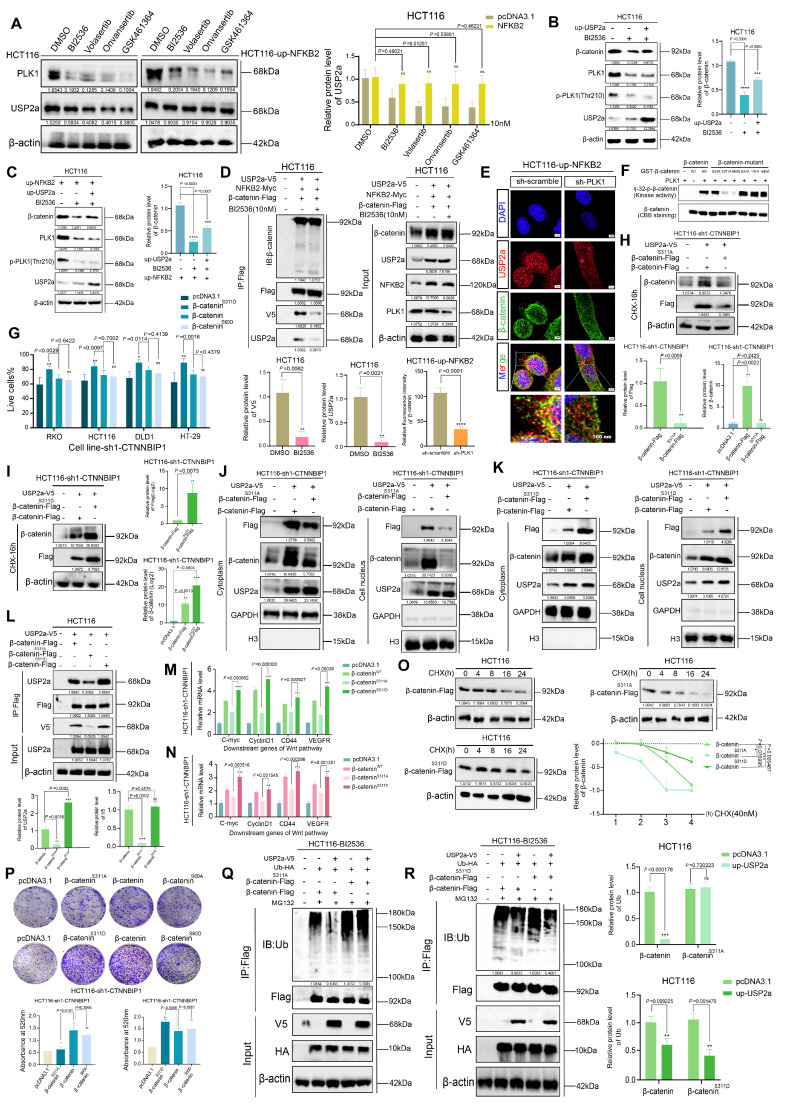
** PLK1-mediated phosphorylation of β-catenin at Ser311 facilitates USP2a recruitment**. **(A)** NFKB2 overexpression rescues USP2a protein levels in HCT116 cells following PLK1 inhibition (10 nM, 12 h) and the recovery period. **(B)** USP2a overexpression partially restored β-catenin protein levels in PLK1-inhibited HCT116 cells. **(C)** Combined NFKB2 and USP2a overexpression partially rescued β-catenin expression in PLK1-inhibited HCT116 cells. **(D)** Coimmunoprecipitation demonstrated that PLK1 inhibition disrupted the USP2a-β-catenin interaction even in NFKB2-overexpressing HCT116 cells. **(E)** Confocal microscopy revealed reduced USP2a fluorescence intensity in PLK1-knockdown HCT116 cells despite NFKB2 overexpression. Scale bar: 2 μm. **(F)** *An in vitro* kinase assay revealed that PLK1 phosphorylates β-catenin at Ser60 and Ser311 but not at degradation-related sites (Ser33, Ser37, Thr41, and Ser45). **(G)** A proliferation assay in β-catenin-knockdown HCT116 cells revealed that the β-catenin-S311D mutant promoted greater growth enhancement than did the wild-type or S60D mutant. **(H-I)** Protein stability analysis demonstrated that β-catenin-S311A exhibited accelerated degradation **(H)**, whereas β-catenin-S311D showed enhanced stability **(I)** in USP2a-overexpressing cells treated with CHX (40 nM). **(J-K)** Subcellular fractionation revealed that β-catenin-S311A displays impaired nuclear accumulation **(J)**, whereas β-catenin-S311D shows increased nuclear localization **(K)** in USP2a-overexpressing cells. **(L)** Coimmunoprecipitation revealed increased binding of USP2a to β-catenin-S311D and reduced interaction with β-catenin-S311A compared with those of wild-type β-catenin. **(M-N)** qPCR analysis revealed that β-catenin-S311D more potently activated Wnt target genes (c-Myc, Cyclin D1, CD44, and VEGFR) than did the wild-type protein, whereas S311A showed reduced activation in two independent β-catenin-knockdown HCT116 cell lines. **(O)** β-catenin-S311D has an extended protein half-life, whereas S311A has decreased stability in CHX chase assays. **(P)** Colony formation assays confirmed that β-catenin-S311D enhances, while S311A impairs, clonogenic growth in β-catenin-knockdown HCT116 cells. **(Q-R)** Ubiquitination assays demonstrated that USP2a overexpression fails to rescue β-catenin-S311A ubiquitination **(Q)** but effectively reduces β-catenin-S311D ubiquitination **(R)** in PLK1-inhibited cells.

While our data established that PLK1 transcriptionally upregulates USP2a via NFKB2 to promote β-catenin deubiquitination, we hypothesized that PLK1 might also directly regulate the β-catenin-USP2a interaction through phosphorylation. In support of these findings, the overexpression of NFKB2 in PLK1-inhibited cells fully rescued the USP2a protein level (Figure 6A, S6A), yet the overexpression of USP2a itself only partially restored the β-catenin level under the same conditions (Figure 6B, C). This discrepancy suggested an additional, USP2a-independent role for PLK1 kinase activity in β-catenin regulation.

We therefore asked whether active PLK1 is required for USP2a-β-catenin complex formation. Co-IP experiments in NFKB2-overexpressing cells revealed that PLK1 inhibition severely impaired the USP2a-β-catenin interaction, reducing complex formation to merely 18% of the control level (Figure 6D). Consistently, confocal microscopy confirmed defective β-catenin nuclear translocation upon PLK1 knockdown (Figure 6E). These results indicate that PLK1 kinase activity is indispensable for orchestrating the functional interplay between USP2a and β-catenin.

To identify specific phosphorylation events, we employed AlphaFold-based prediction, which highlighted β-catenin Ser60 and Ser311 as potential PLK1 target sites. *In vitro* kinase assays using purified PLK1 demonstrated that mutation of either Ser60 or Ser311, but not the canonical degradation-related sites (Ser33, Ser37, Thr41, and Ser45), abolished PLK1-mediated β-catenin phosphorylation (Figure 6F).

We next characterized the functional hierarchy of these sites. In β-catenin-knockdown CRC cells (Figure S6B-E), reconstitution with phosphomimetic mutants revealed that β-catenin-S311D dramatically enhanced cell proliferation and colony formation, outperforming both wild-type (WT) and β-catenin-S60D (Figure 6G, P, S6F). Conversely, the phospho-dead β-catenin-S311A mutant, but not S60A, potently suppressed these oncogenic phenotypes (Figure S6G). Immunofluorescence further demonstrated that β-catenin-S311D, but not S60D, enhanced the nuclear localization and increased the intensity of proliferation markers (p-H3 and KI67), whereas S311A diminished the intensity (Figure 6H-N, S6H, S6I).

Mechanistically, the β-catenin-S311D mutant presented a prolonged half-life (Figure 6I, O) and increased nuclear accumulation (Figure 6K), whereas the S311A mutant was rapidly degraded (Figure 6H, O) and retained in the cytoplasm (Figure 6J). Crucially, co-IP assays demonstrated that β-catenin-S311D bound USP2a more robustly, whereas β-catenin-S311A had a weakened interaction (Figure 6L). The functional culmination of this phospho-primed binding was revealed via ubiquitination assays: USP2a overexpression rescued β-catenin ubiquitination in the presence of WT or S311D β-catenin but failed to do so when β-catenin was locked into the S311A state (Figure 6Q, R).

In summary, we elucidate a dual mechanism whereby PLK1 stabilizes β-catenin: first, by activating the NFKB2-USP2a transcriptional axis to provide the deubiquitination machinery; second, by directly phosphorylating β-catenin at Ser311, which acts as a molecular “switch” to support its efficient recruitment to and stabilization by USP2a. This coordinated posttranslational and transcriptional regulation ensures robust activation of the Wnt/β-catenin pathway in colorectal cancer.

### PLK1 drives tumor growth and inflammatory angiogenesis *in vivo* via the NFKB2-USP2a-β-catenin axis

**Figure 7:**
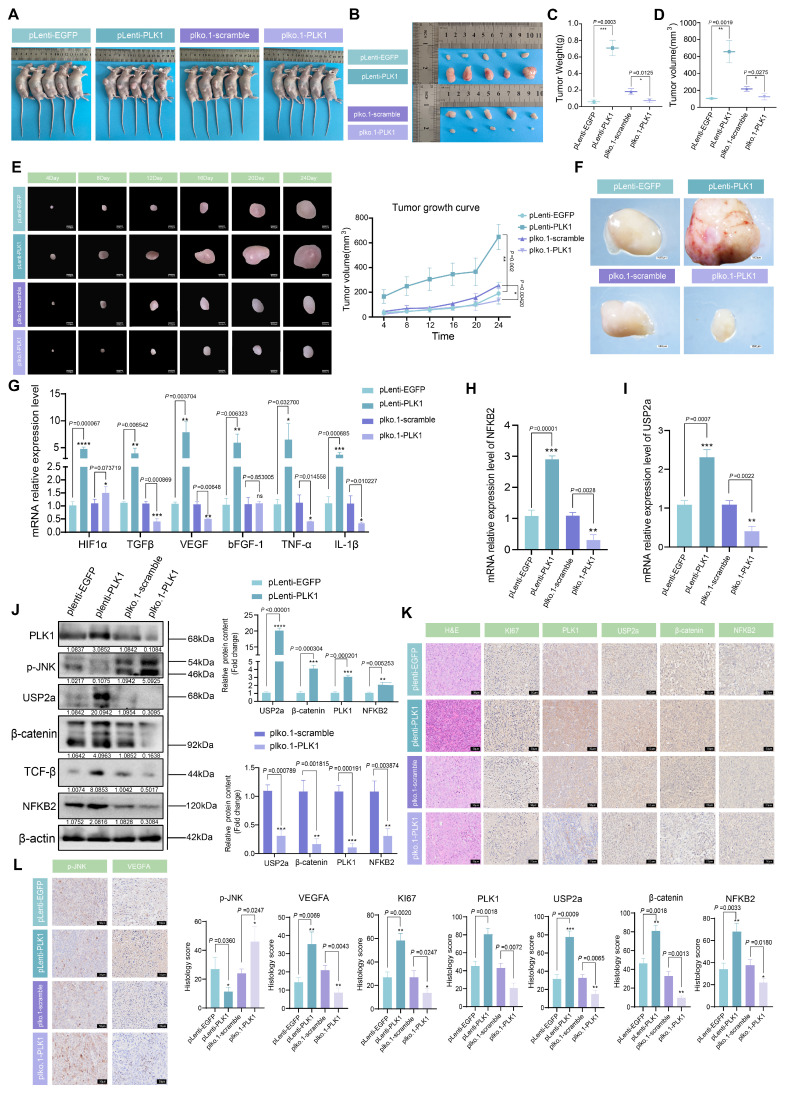
** PLK1 promotes tumor growth in CRC xenograft models. (A)** Schematic of the xenograft tumor experiment in nude mice. **(B)** Representative images of dissected tumors from nude mice at the experimental endpoint (day 28 postinoculation). **(C)** Tumor weights from different experimental groups. The data are presented as the means ± SEMs. **(D)** Tumor volumes were calculated via the formula V = π/6 × L × W² (mm³) on the basis of final length and width measurements. **(E)** Tumor growth curves showing volume measurements every 4 days for the PLK1-overexpressing and PLK1-knockdown groups. The data represent the means ± SEMs. **(F)** Macroscopic examination revealing enhanced capillary network formation on the surface of PLK1-overexpressing tumors. **(G)** qPCR analysis of inflammatory factors (HIF1α, TGFβ, bFGF-1, TNF-α, and IL-1β) in xenograft tumors. The data are presented as the means ± SEMs. **(H)** NFKB2 mRNA levels in xenograft tumors from different experimental groups. The data represent the means ± SEMs. **(I)** USP2a transcript levels in xenograft tumors across experimental groups. The data are presented as the means ± SEMs. **(J)** Western blot analysis of PLK1, NFKB2, USP2a, β-catenin, p-JNK, and TGF-β protein expression in xenograft tumors. **(K)** Immunohistochemical staining of H&E, Ki67, PLK1, USP2a, β-catenin, and NFKB2 in xenograft tumor sections. Scale bar: 20 μm. **(L)** Immunohistochemical analysis of p-JNK and VEGFA expression in xenograft tumors. Scale bar: 20 μm.

Our *in vitro* findings established that PLK1 stabilizes β-catenin to sustain Wnt pathway activation and promote CRC proliferation. To validate the biological significance of this axis in a physiological context, we established a nude mouse xenograft model using HCT116 cells with stable PLK1 overexpression (HCT116-pLenti-PLK1) or knockdown (HCT116-pLKO.1-PLK1) (Figure 7A).

Monitoring of tumor growth indicated that PLK1 overexpression significantly accelerated tumor development, leading to a 2.8-fold increase in final tumor volume compared to the control. Conversely, PLK1 knockdown suppressed tumor growth by 64.5% (Figure 7E). At the experimental endpoint (day 28), the average tumor weight in the PLK1-overexpressing group (0.754 ± 0.15 g) was 58.5% greater than that in the control group (0.129 ± 0.11 g), while that in the PLK1-knockdown group (0.131 ± 0.09 g) was significantly lower (Figure 7B-D).

To further substantiate the therapeutic importance of targeting PLK1, we treated HCT116 xenograft-bearing mice with the four PLK1 inhibitors used in our study. The inhibitor-treated groups showed significantly reduced tumor growth and smaller tumor sizes and weights compared to the control group (Figure S7A-C, E). This pharmacologic evidence perfectly corroborated the genetic data.

Macroscopic examination of dissected tumors revealed denser capillary networks on the surface of PLK1-overexpressing tumors, indicative of enhanced angiogenesis—a phenotype that was absent in both the PLK1-knockdown and inhibitor-treated groups (Figure 7F, S7D). Given the link between tumor progression and inflammation, we analyzed the expression of inflammatory mediators. qPCR analysis of tumor RNA revealed that PLK1 overexpression upregulated the mRNA levels of HIF1α, TGFβ, bFGF-1, TNF-α, and IL-1β, whereas PLK1 knockdown or inhibition had the opposite effect (Figure 7G, S7I). Furthermore, the mRNA levels of NFKB2 and USP2a were positively correlated with PLK1 expression in tumors (Figure 7H, I), confirming the conservation of the PLK1-NFKB2-USP2a transcriptional axis *in vivo*.

Western blot and immunohistochemical (IHC) analyses of the tumor tissues provided mechanistic confirmation. The protein levels of NFKB2, USP2a, β-catenin, and the inflammatory factor TGFβ were elevated in PLK1-overexpressing tumors and reduced upon PLK1 knockdown or inhibition (Figure 7J, S7H). IHC staining revealed that PLK1-overexpressing tumors presented deeper nuclear staining, tighter cell packing, and increased Ki67 positivity, all of which are indicative of heightened proliferative activity. These features were diminished in the PLK1-knockdown and inhibitor-treated groups (Figure 7K, S7F). Consistent with the proangiogenic phenotype, PLK1-overexpressing tumors exhibited increased VEGFA and p-JNK staining (Figure 7L, S7G).

In summary, our *in vivo* data provide conclusive genetic and pharmacologic evidence that PLK1 is a potent driver of CRC tumor growth. It functions by activating the NFKB2-USP2a-β-catenin signaling axis, which in turn promotes a tumor microenvironment characterized by increased inflammation and angiogenesis, thereby confirming the therapeutic potential of PLK1 inhibition in colorectal cancer.

## Discussion

**Figure 8:**
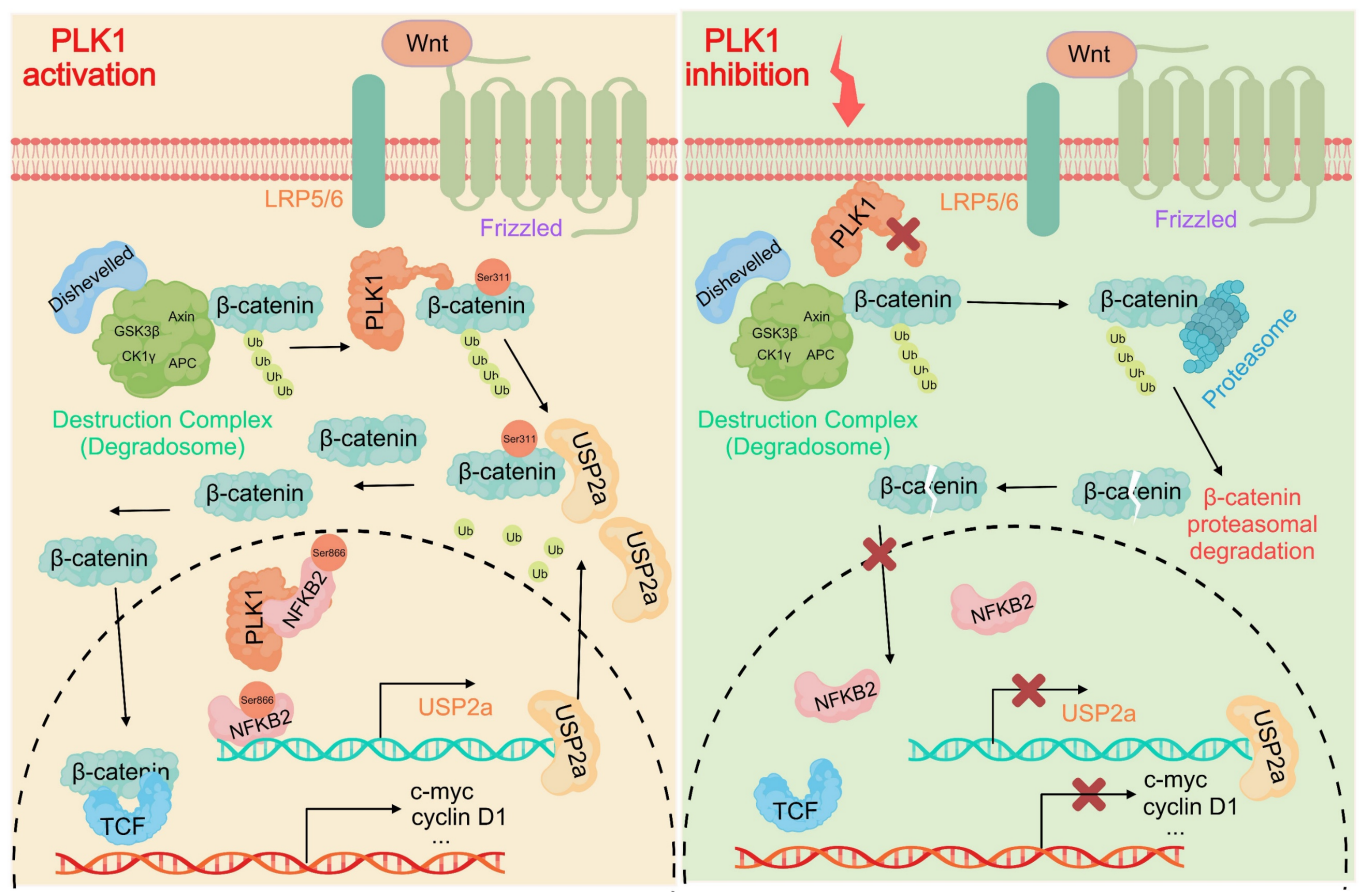
** PLK1 activates the USP2a/β-catenin signaling axis to drive colorectal carcinogenesis.** In colorectal cancer cells, PLK1 orchestrates sustained Wnt/β-catenin activation through two convergent mechanisms: transcriptional induction via PLK1-mediated phosphorylation of NFKB2 at Ser866/870, which activates *USP2a* gene expression and elevates USP2a protein levels; coupled with direct phosphorylation of β-catenin at Ser311 that recruits USP2a to suppress β-catenin ubiquitination and degradation. Collectively, these coordinated actions promote malignant progression by enabling nuclear accumulation of β-catenin and persistent activation of downstream oncogenic targets.

Our study elucidates a previously unrecognized dual-axis mechanism through which PLK1 orchestrates Wnt/β-catenin hyperactivation in colorectal cancer. We demonstrated that PLK1 coordinately regulates β-catenin stability through two synergistic pathways: direct phosphorylation at Ser311 to promote USP2a-mediated deubiquitination and NFKB2-driven transcriptional upregulation of USP2a (Figure 8). This self-reinforcing circuit sustains nuclear β-catenin accumulation and drives CRC proliferation, extending PLK1's function beyond its canonical mitotic roles to position it as a critical nexus between cell cycle progression and Wnt signaling addiction.

The identification of β-catenin-Ser311 phosphorylation as a molecular prerequisite for USP2a recruitment provides mechanistic insight into the incomplete rescue of β-catenin stability observed upon USP2a overexpression in PLK1-inhibited cells. This phospho-dependent complex assembly represents a fundamental divergence from the role of PLK1 in non-small cell lung cancer, where Ser311 phosphorylation stabilizes β-catenin independently of USP2a. Such tissue-specific regulatory wiring may reflect the unique reliance of CRC on USP2a, a deubiquitinase frequently amplified in colorectal malignancies, highlighting the imperative for context-aware therapeutic targeting.

In addition to protein‒protein interactions, our work revealed the capacity of PLK1 to amplify Wnt signaling through NFKB2-dependent transcriptional programming. PLK1-mediated phosphorylation of NFKB2 at Ser866/870 enhances its transactivation potential toward the USP2a promoter, establishing a feed-forward loop wherein stabilized β-catenin further amplifies NFKB2 expression through β-catenin/TCF4 binding to the NFKB2 promoter 33,34. While NF-κB/Wnt crosstalk has been documented in other cancers, our identification of PLK1 as the initiator of this cascade in CRC represents a significant advance 35. This transcriptional axis may additionally underpin the role of PLK1 in chemoresistance, as NFKB2-mediated USP2a upregulation stabilizes known 5-FU resistance markers, which is consistent with elevated PLK1 expression in our 5-FU-resistant models.

Therapeutically, this dual-axis mechanism offers a strategic solution to persistent challenges in Wnt pathway inhibition. Conventional β-catenin antagonists often cause dose-limiting gastrointestinal toxicity by disrupting Wnt signaling in normal intestinal stem cells, whereas single-agent PLK1 inhibitors may trigger compensatory pathway reactivation. Our data suggest that PLK1 blockade concurrently disrupts β-catenin stability at two critical nodes: USP2a transcription (via NFKB2 suppression) and phospho-primed deubiquitination (via kinase inhibition). This coordinated disruption may circumvent toxicity concerns, as PLK1 is preferentially overexpressed in tumors compared with quiescent intestinal stem cells. In support of this premise, recent combinatorial studies have demonstrated that volasertib synergizes with PRI-724 to suppress CRC xenograft growth without exacerbating intestinal damage—a synergistic effect potentially rooted in our identified mechanism.

Our findings also open several promising research avenues. The established stabilization of PD-L1 by USP2a in CRC suggests that PLK1 inhibition, through USP2a downregulation, could enhance anti-PD-L1 efficacy by remodeling immunosuppressive microenvironments—a hypothesis supported by single-cell RNA-seq studies 36,37. Additionally, emerging evidence indicates that USP2a stabilizes metabolic enzymes such as hexokinase 2, potentially coupling Wnt activation to glycolytic flux and revealing novel metabolic dependencies in CRC.

Several limitations merit consideration for future investigations. While NFKB2 has emerged as central to USP2a transcription, other PLK1 effectors (e.g., STAT3) may contribute, particularly in inflammatory CRC subtypes. The functional distinction between USP2 splice variants (USP2a/2b) also requires clarification, as does the influence of the native colonic microenvironment on this regulatory axis. To address these questions, we propose two priority investigations: genotype-directed therapy in which PLK1 inhibitors are tested in APC-mutant versus wild-type CRC models and the development of USP2a-directed PROTACs utilizing PLK1-binding ligands to achieve dual-pathway inhibition.

## Conclusion

In conclusion, our work provides a unifying framework for understanding β-catenin signaling plasticity in CRC. By elucidating PLK1's dual regulation through phosphorylation-dependent recruitment to USP2a and transcriptional reinforcement of USP2a expression, we not only redefine PLK1 as a master coordinator of oncogenic signaling but also present a compelling rationale for toxicity-sparing combinatorial regimens targeting aggressive colorectal malignancies.

## Materials and Methods

### Cell culture

Colorectal cancer cell lines (HCT116, HT-29, DLD1, RKO, SW480, LoVo, Caco-2) and normal colonic epithelial cells (NCM460) were cultured in RPMI-1640 medium (Gibco, 11875) with 10% fetal bovine serum (FBS, Gibco, 10270) and 1% penicillin-streptomycin (Beyotime, C0222). Cells were cultured at 37°C in a 5% CO₂ humidified incubator (Thermo, HERAcell 150i). Cell authentication was performed via STR profiling (Microread, Beijing). Cells were subcultured using 0.25% trypsin-EDTA (Beyotime, C0201) at 80% confluence for up to 20 passages.

### RNA extraction and RT‒qPCR

Total RNA was isolated with TRIzol reagent (Invitrogen, 15596026), followed by chloroform phase separation. The RNA pellets were rinsed with 75% ethanol and dissolved in nuclease-free water. The RNA concentration and purity, indicated by an A260/A280 ratio of 1.8-2.0, were assessed using a NanoDrop 2000 spectrophotometer (Thermo). cDNA was synthesized using 1 μg of RNA and HiScript II Q RT SuperMix (Vazyme, R223). Quantitative PCR was conducted using ChamQ SYBR Master Mix (Vazyme, Q331) on a QuantStudio 6 instrument (Applied Biosystems) following standard protocol: initial denaturation at 95°C for 30 seconds, followed by 40 cycles of 95 °C for 10 seconds and 60 °C for 30 seconds. *GAPDH* served as an endogenous control. Refer to Supplementary Table 1 for the primer sequences.

### Antibodies and reagents

The primary antibodies utilized included: p-PLK1 (ABclonal), p-PLK1 (Thr210) (ABclonal), Flag (Positive), H3 (Positive), β-catenin (Positive Energy), β-catenin (Abways), Ub (Proteintech), USP2a (BioVision), V5 (Positive), NFKB2 (Vanguard), p-NFKB2 (Ser866/870) (ABclonal), VEGFA (ABclonal), p-JNK (ABclonal), and TGF-β (Positive). The secondary antibodies utilized were HRP-conjugated anti-rabbit/mouse IgG from CST.

The following inhibitors were used: BI2536 (PLK1 inhibitor, Selleckchem, S1109; 10 nM), volasertib (PLK1 inhibitor, Selleckchem, S2235; 10 nM), onvansertib (PLK1 inhibitor, Selleckchem, S7255; 10 nM), GSK461324 (PLK1 inhibitor, Selleckchem, S2193; 10 nM), and Wnt-agonist 1 (Wnt activator, Selleckchem, S8178; 2 nM).

### Western blotting

The cells were lysed in RIPA buffer (Beyotime, P0013B) containing 1× protease/phosphatase inhibitor cocktail (MedChemExpress, HY-K0010). The lysates were centrifuged at 12,000 × g for 15 minutes at 4 °C. The protein concentration was determined via a BCA assay (Beyotime, P0010). Proteins (20-40 μg) were resolved using 8-12% SDS-PAGE and subsequently transferred onto PVDF membranes (Millipore, IPFL00010). Membranes were blocked with 5% BSA (Solaibio, A8010) in TBST for 1 hour, followed by overnight incubation with primary antibodies at 4°C and a 1-hour incubation with HRP-conjugated secondary antibodies at room temperature. The signals were developed via ECL Prime (GE Healthcare, RPN2232) and captured via ChemiDoc MP (Bio-Rad).

### Coimmunoprecipitation (Co-IP)

Cell lysates (500 μg) were cleared using protein A/G magnetic beads (Thermo, 88802) for an hour, followed by an overnight incubation with 2 μg of primary antibody at 4 °C. Antibody complexes were isolated using beads for 2 hours, followed by four washes with ice-cold lysis buffer. The bound proteins were then eluted in 2× Laemmli buffer at 95 °C for 10 minutes. Western blot analysis was conducted on both input and IP samples.

### Colony formation assay

The cells (500/well) were seeded in 6-well plates (Corning) and cultured for 14 days, and the medium was changed every 3 days. Colonies were fixed with methanol for 30 min, stained with 0.5% crystal violet (Beyotime, C0121) for 20 min, and counted under a stereomicroscope (Olympus, SZX7). Colonies with > 50 cells were quantified.

### Cell proliferation assay

Cell viability was assessed using a CCK-8 kit (Dojindo, CK04). The cells (3×10³/well) were seeded in 96-well plates. CCK-8 reagent (10 μL) was added to each well at 0, 24, 48, and 72 hours. The absorbance at 450 nm was recorded using a BioTek Synergy H1 microplate reader following a 2-hour incubation at 37 °C.

### Wound healing assay

Confluent monolayers in 12-well plates were scratched using a sterile 200 μL pipette tip. Detached cells were eliminated by washing with PBS. Migration was observed at 0 and 24 hours using a Nikon Eclipse Ts2 inverted microscope. The percentage of gap closure was quantified via ImageJ (v1.53).

### Transwell invasion assay

Transwell inserts (Corning, 3422) were coated with Matrigel (BD Biosciences, 356234) diluted 1:8 in serum-free medium and allowed to solidify for 4 hours at 37 °C. Cells deprived of serum (2×10⁵ in 200 μL of medium without serum) were placed in the upper chambers. The lower chambers contained 600 μL of medium supplemented with 20% FBS as a chemoattractant. After 24 h, noninvaded cells were removed with cotton swabs. Cells that were invaded were fixed using methanol, stained with 0.1% crystal violet, and counted across five random fields per insert at 200× magnification.

### Dual-luciferase reporter assay

Cells in 24-well plates were cotransfected using Lipofectamine 3000 (Invitrogen, L3000001) with 400 ng of either wild-type or mutant pGL3-USP2a-promoter and 40 ng of pRL-TK (Renilla control). Cells were lysed after 48 hours, and firefly/Renilla luciferase activities were assessed using the Dual-Luciferase Reporter Assay System (Promega, E1910) on a GloMax Navigator (Promega). Firefly luminescence was normalized to Renilla luminescence.

### Subcellular fractionation

Nuclear and cytoplasmic proteins were isolated using the NE-PER Kit (Thermo, 78833) following the manufacturer's instructions. Briefly, the cells were lysed in CER-I buffer, vortexed, incubated on ice, and then mixed with CER-II. The supernatant was obtained by centrifuging the cytoplasmic fraction at 16,000 × g for 5 minutes. The nuclear pellets were resuspended in NER buffer and vortexed. Fraction purity was verified by Lamin B1 (nuclear) and α-Tubulin (cytoplasmic) markers.

### Lentiviral transduction

Lentiviral shRNAs targeting PLK1 and the overexpression vector (pLenti-PLK1) were cotransfected with the packaging plasmids psPAX2 (Addgene, #12260) and pMD2. G (Addgene, #12259) into 293T cells via PEI (Polysciences, 24765). Viral supernatants were collected at 48 and 72 hours, concentrated using PEG-it (SBI, LV810A-1), and introduced into CRC cells with 8 μg/ml polybrene (Sigma, TR1003). Stable lines were established by selecting with 2 μg/ml puromycin (MedChemExpress, HY-B1743A) over a period of 7 days.

### Immunofluorescence

Cells cultured on glass coverslips were fixed using 4% paraformaldehyde (Beyotime, P0098) for 15 minutes, then permeabilized with 0.1% Triton X-100 (Beyotime, ST795) for 10 minutes, and blocked with 5% BSA for 1 hour. Primary antibodies were incubated overnight at 4 °C, followed by a 1-hour incubation with Alexa Fluor 488/594-conjugated secondary antibodies (Invitrogen, A-11008/A-11032) at room temperature. Nuclei were stained using DAPI (Beyotime, C1006). Images were acquired via an LSM 900 confocal microscope (Zeiss) with a 63× oil objective and processed with ZEN software.

### Xenograft tumor model

The Sichuan University Animal Ethics Committee (Approval No.) sanctioned all animal procedures. SYXK2023-189) following NIH guidelines. Four-week-old female BALB/c nude mice (Vital River) were maintained in a specific pathogen-free environment. HCT116 cells (2×10⁶) with stable expression of shPLK1, oePLK1, or a control vector were subcutaneously injected into the right flank of subjects (n = 5 per group) using 100 μL of a PBS/Matrigel (1:1) mixture. Tumor volumes were assessed biweekly using calipers and calculated using the formula V = π/6 × L × W² (mm³). The mice were sacrificed on day 28. Tumors were weighed, photographed, and processed for IHC/qPCR.

### Statistical analysis

Data from ≥ 3 independent experiments are presented as the means ± SDs. Statistical significance was assessed using a two-tailed Student's t-test for two groups or a one-way ANOVA with Tukey's post hoc test for multiple groups, conducted with GraphPad Prism 8.0. Significance levels were denoted as follows: *p < 0.05, **p < 0.01, ***p < 0.001, ****p < 0.0001.

## Supplementary Material

Supplementary figures and table.

## Abbreviations

CRC: colorectal cancer
PLK1: polo-like kinase 1
USP2a: ubiquitin-specific protease 2a
NFKB2: human nuclear factor-κB2
NSCLC: non-small cell lung cancer
ESCC: esophageal squamous cell carcinoma
GSK-3β: glycogen synthase kinase-3β
APC: adenomatous polyposis coli
CK1α: casein kinase 1α
DUBs: deubiquitinating enzymes
PD-L1: programmed death ligand-1
KD: kinase domain
PBD: polo-box domain
FZD: Frizzled
Co-IP: coimmunoprecipitation
CHX: cycloheximide
STAT3: Signal transducer and activator of transcription 3
H&E: hematoxylin and eosin

## References

[1] Cho YH, Ro EJ, Yoon JS, Mizutani T, Kang DW, Park JC (2020). 5-FU promotes stemness of colorectal cancer via p53-mediated WNT/β-catenin pathway activation. Nat Commun.

[2] Yang D, Li Q, Shang R, Yao L, Wu L, Zhang M (2020). WNT4 secreted by tumor tissues promotes tumor progression in colorectal cancer by activation of the Wnt/β-catenin signalling pathway. J Exp Clin Cancer Res.

[3] Lettini G, Sisinni L, Condelli V, Matassa DS, Simeon V, Maddalena F (2016). TRAP1 regulates stemness through Wnt/β-catenin pathway in human colorectal carcinoma. Cell Death Differ.

[4] Liu X, Su K, Sun X, Jiang Y, Wang L, Hu C (2021). Sec62 promotes stemness and chemoresistance of human colorectal cancer through activating Wnt/β-catenin pathway. J Exp Clin Cancer Res.

[5] Jung YS, Park JI (2020). Wnt signaling in cancer: therapeutic targeting of Wnt signaling beyond β-catenin and the destruction complex. Exp Mol Med.

[6] Yu F, Yu C, Li F, Zuo Y, Wang Y, Yao L (2021). Wnt/β-catenin signaling in cancers and targeted therapies. Signal Transduct Target Ther.

[7] Kim DE, Shin SB, Kim CH, Kim YB, Oh HJ, Yim H (2023). PLK1-mediated phosphorylation of beta-catenin enhances its stability and transcriptional activity for extracellular matrix remodeling in metastatic NSCLC. Theranostics.

[8] He S, Tang S (2020). WNT/β-catenin signaling in the development of liver cancers. Biomed Pharmacother.

[9] Wang R, Liu J, Li K, Yang G, Chen S, Wu J (2021). An SETD1A/Wnt/β-catenin feedback loop promotes NSCLC development. J Exp Clin Cancer Res.

[10] Ng VH, Spencer Z, Neitzel LR, Nayak A, Loberg MA, Shen C (2023). The USP46 complex deubiquitylates LRP6 to promote Wnt/β-catenin signaling. Nat Commun.

[11] Liu L, Zhang Y, Wong CC, Zhang J, Dong Y, Li X (2018). RNF6 Promotes Colorectal Cancer by Activating the Wnt/β-Catenin Pathway via Ubiquitination of TLE3. Cancer Res.

[12] Tomala MD, Magiera-Mularz K, Kubica K, Krzanik S, Zieba B, Musielak B (2018). Identification of small-molecule inhibitors of USP2a. Eur J Med Chem.

[13] Zhao Y, Wang X, Wang Q, Deng Y, Li K, Zhang M (2018). USP2a Supports Metastasis by Tuning TGF-β Signaling. Cell Rep.

[14] Lin DC, Zhang Y, Pan QJ, Yang H, Shi ZZ, Xie ZH (2011). PLK1 Is transcriptionally activated by NF-κB during cell detachment and enhances anoikis resistance through inhibiting β-catenin degradation in esophageal squamous cell carcinoma. Clin Cancer Res.

[15] Yu JE, Kim SO, Hwang JA, Hong JT, Hwang J, Soung NK (2021). Phosphorylation of β-catenin Ser60 by polo-like kinase 1 drives the completion of cytokinesis. EMBO Rep.

[16] Kundu M, Greer YE, Lobanov A, Ridnour L, Donahue RN, Ng Y TRAIL induces cytokine production via the NFkB2 pathway promoting neutrophil chemotaxis and neutrophil-mediated immune-suppression in triple negative breast cancer cells. Cancer Lett. 2025: 217692.

[17] Li Y, He X, Wang S, Shu HB, Liu Y (2013). USP2a positively regulates TCR-induced NF-κB activation by bridging MALT1-TRAF6. Protein Cell.

[18] Ahn DH, Barzi A, Ridinger M, Samuëlsz E, Subramanian RA, Croucher PJP (2024). Onvansertib in Combination with FOLFIRI and Bevacizumab in Second-Line Treatment of KRAS-Mutant Metastatic Colorectal Cancer: A Phase Ib Clinical Study. Clin Cancer Res.

[19] Lu Y, Sun Y, Zhang J, Kong M, Zhao Z, Sun B (2024). The deubiquitinase USP2a promotes tumor immunosuppression by stabilizing immune checkpoint B7-H4 in lung adenocarcinoma harboring EGFR-activating mutants. Cancer Lett.

[20] Ciardo D, Haccard O, Narassimprakash H, Cornu D, Guerrera IC, Goldar A (2021). Polo-like kinase 1 (Plk1) regulates DNA replication origin firing and interacts with Rif1 in Xenopus. Nucleic Acids Res.

[21] Conti D, Verza AE, Pesenti ME, Cmentowski V, Vetter IR, Pan D (2024). Role of protein kinase PLK1 in the epigenetic maintenance of centromeres. Science.

[22] Beck J, Maerki S, Posch M, Metzger T, Persaud A, Scheel H (2013). Ubiquitylation-dependent localization of PLK1 in mitosis. Nat Cell Biol.

[23] Zhou Q, Chen S, Lu M, Luo Y, Wang G, Xiao Y (2019). EFEMP2 suppresses epithelial-mesenchymal transition via Wnt/β-catenin signaling pathway in human bladder cancer. Int J Biol Sci.

[24] Zhang K, Guo Y, Wang X, Zhao H, Ji Z, Cheng C (2017). WNT/β-Catenin Directs Self-Renewal Symmetric Cell Division of hTERT(high) Prostate Cancer Stem Cells. Cancer Res.

[25] Lu FI, Sun YH, Wei CY, Thisse C, Thisse B (2014). Tissue-specific derepression of TCF/LEF controls the activity of the Wnt/β-catenin pathway. Nat Commun.

[26] Cao Y, Geng J, Wang X, Meng Q, Xu S, Lang Y (2022). RNA-binding motif protein 10 represses tumor progression through the Wnt/β- catenin pathway in lung adenocarcinoma. Int J Biol Sci.

[27] Voorneveld PW, Kodach LL, Jacobs RJ, van Noesel CJ, Peppelenbosch MP, Korkmaz KS (2015). The BMP pathway either enhances or inhibits the Wnt pathway depending on the SMAD4 and p53 status in CRC. Br J Cancer.

[28] Yu JE, Kim SO, Hwang JA, Hong JT, Hwang J, Soung NK (2021). Phosphorylation of beta-catenin Ser60 by polo-like kinase 1 drives the completion of cytokinesis. EMBO Rep.

[29] Malki A, ElRuz RA, Gupta I, Allouch A, Vranic S, Al Moustafa AE (2020). Molecular Mechanisms of Colon Cancer Progression and Metastasis: Recent Insights and Advancements. Int J Mol Sci.

[30] Zhu GX, Gao D, Shao ZZ, Chen L, Ding WJ, Yu QF (2021). Wnt/beta-catenin signaling: Causes and treatment targets of drug resistance in colorectal cancer (Review). Mol Med Rep.

[31] Xu Q, Liu M, Zhang F, Liu X, Ling S, Chen X (2021). Ubiquitin-specific protease 2 regulates Ang Ⅱ-induced cardiac fibroblasts activation by up-regulating cyclin D1 and stabilizing beta-catenin in vitro. J Cell Mol Med.

[32] Kitamura H, Hashimoto M (2021). USP2-Related Cellular Signaling and Consequent Pathophysiological Outcomes. Int J Mol Sci.

[33] Zhu X, Gu J, Qian H (2018). Esculetin Attenuates the Growth of Lung Cancer by Downregulating Wnt Targeted Genes and Suppressing NF-κB. Arch Bronconeumol (Engl Ed).

[34] Oliva-Vilarnau N, Beusch CM, Sabatier P, Sakaraki E, Tjaden A, Graetz L (2024). Wnt/β-catenin and NFκB signaling synergize to trigger growth factor-free regeneration of adult primary human hepatocytes. Hepatology.

[35] Zhang Z, Cheng L, Li J, Qiao Q, Karki A, Allison DB (2022). Targeting Plk1 Sensitizes Pancreatic Cancer to Immune Checkpoint Therapy. Cancer Res.

[36] Wang W, Li M, Ponnusamy S, Chi Y, Xue J, Fahmy B (2020). ABL1-dependent OTULIN phosphorylation promotes genotoxic Wnt/β-catenin activation to enhance drug resistance in breast cancers. Nat Commun.

[37] Kuang Z, Liu X, Zhang N, Dong J, Sun C, Yin M (2023). USP2 promotes tumor immune evasion via deubiquitination and stabilization of PD-L1. Cell Death Differ.

