# Supplementary material for: PLK1 stabilizes β-catenin to drive colorectal carcinogenesis through NFKB2-mediated transcriptional activation of USP2a and site-specific phosphorylation
Source: Theranostics. 2026 Jan 1;16(7):3488–506. doi: 10.7150/thno.122368 (PMC12846735; doi:10.7150/thno.122368)
Supplement: Supplementary file 1 — Supplementary figures and table. [file thnov16p3488s1.pdf]

Supplemental Materials

PLK1 stabilizes β-catenin to drive colorectal carcinogenesis through NFKB2-mediated transcriptional activation of USP2a and site-specific phosphorylation

Yan Li, Lili Zhao, Jiaqiang Deng, Songmei Lu, Jingyu Du, Chen Chen, Li Zhao, Zhiyun Xu, Wencan Wang, Yundi Wang and Fangdong Zou *

The PDF file includes:

Supplemental Figures 1 to 7

Supplemental Table 1

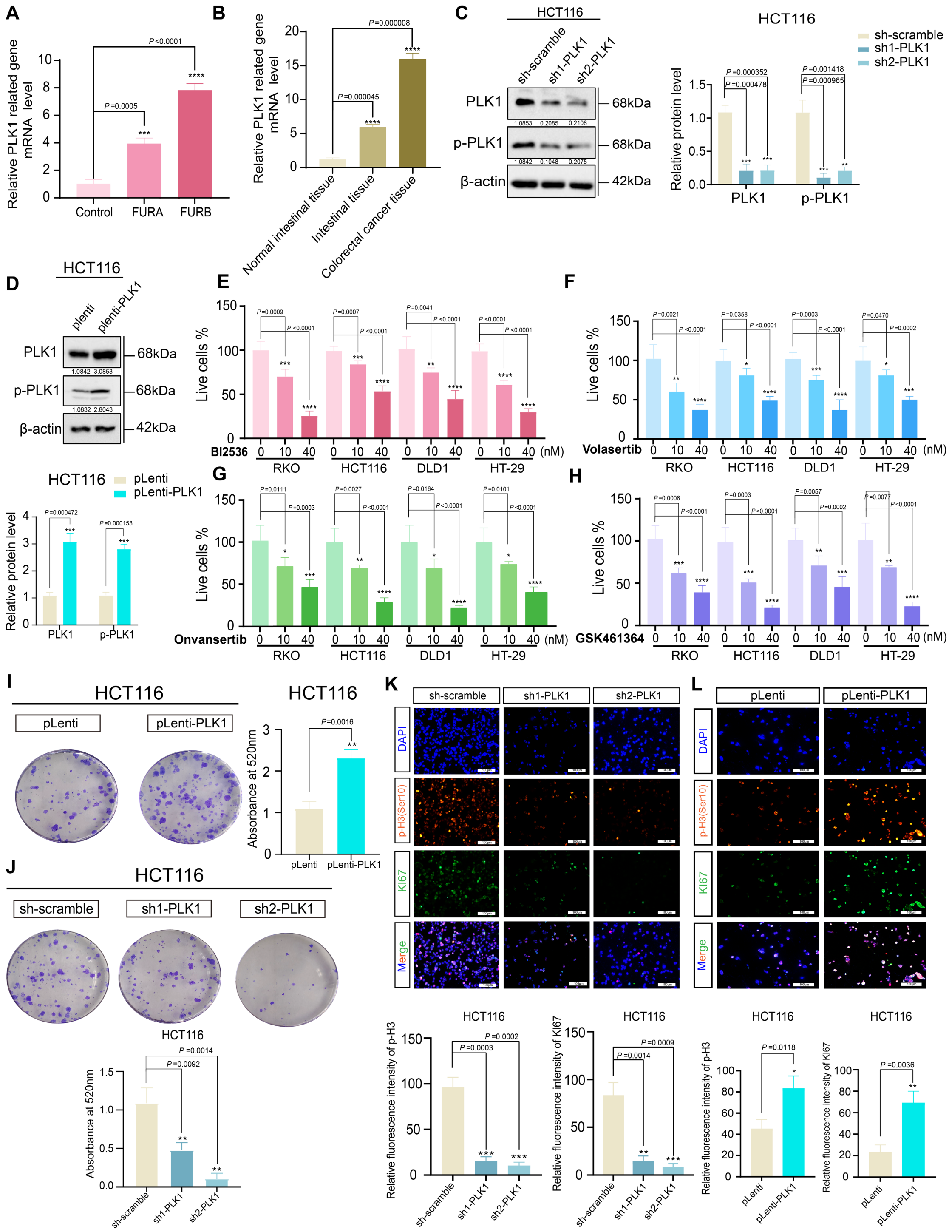

Figure S1. PLK1 is aberrantly overexpressed in CRC and drives malignant phenotypes. **(A)** PLK1 mRNA expression in 5-FU-resistant HCT116 sublines (FURA, 20 μM; FURB, 40 μM) compared with parental cells. **(B)** PLK1 transcript levels in mouse models of intestinal inflammation and colorectal cancer compared with those in normal intestinal tissue. **(C)** Validation of PLK1-knockdown stable cell lines. HCT116 cells were infected with pLKO.1-sh1-PLK1 or pLKO.1-sh2-PLK1 lentivirus and selected with puromycin (1 μg/mL, 14 days). PLK1 and p-PLK1 (Thr210) protein levels

were assessed via Western blotting. **(D)** Validation of the PLK1-overexpressing stable cell line. HCT116 cells were infected with pLenti-PLK1 lentivirus and selected with puromycin (1 μg/mL, 14 days). PLK1 and p-PLK1 (Thr210) expression was confirmed by Western blotting. **(E-H)** Cell viability was assessed by a CCK-8 assay in CRC cell lines (RKO, HCT116, DLD1, and HT-29) treated with PLK1 inhibitors (BI2536, volasertib, onvansertib, and GSK461364) at 10 nM and 40 nM for 24 hours. **(I)** Colony formation assay showing increased colony numbers in PLK1-overexpressing HCT116 cells (HCT116-pLenti-PLK1) after 14 days of culture. **(J)** Reduced colony formation in PLK1-knockdown HCT116 cells (HCT116-pLKO.1-sh1/2-PLK1) after 14 days of culture. **(K)** Immunofluorescence analysis showing decreased KI67 and p-H3 (Ser10) fluorescence intensity in PLK1-knockdown HCT116 cells. **(L)** Increased KI67 and p-H3 (Ser10) fluorescence intensity in PLK1-overexpressing HCT116 cells.

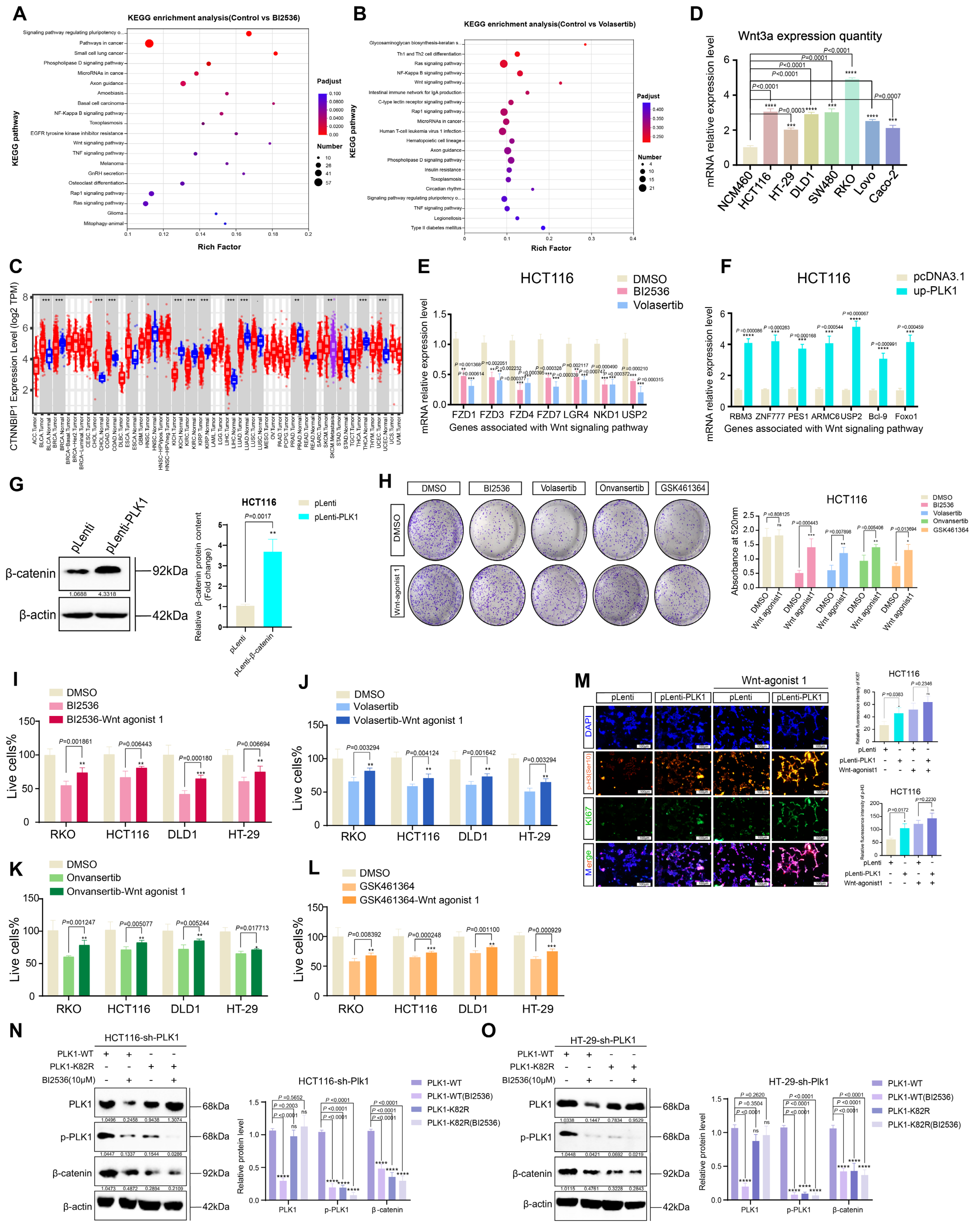

Figure S2. PLK1 activates the Wnt/β-catenin signaling pathway in colorectal cancer. (A-B) KEGG pathway enrichment analysis of HCT116 cells treated with BI2536 (1 nM) or volasertib (1 nM) for 12 hours, which revealed specific enrichment of the Wnt signaling pathway, among other pathways. **(C)** Analysis of β-catenin gene expression across different tumor types from the TIMER database, comparing tumor tissues with adjacent normal tissues. **(D)** Wnt3a mRNA expression levels in CRC cell lines compared with those in normal intestinal epithelial cells. **(E)** qPCR analysis of Wnt/β-catenin downstream genes (FZD1, FZD3, FZD4, FZD7, LGR4, NKD1, and

USP2) in HCT116 cells treated with BI2536 or volasertib (1 nM, 12 hours). **(F)** qPCR analysis of Wnt/β-catenin downstream target genes in HCT116 cells transfected with pcDNA3.1-PLK1 for 24 hours. **(G)** Validation of β-catenin overexpression in lentivirus-infected stable HCT116 cell lines (4-fold overexpression). **(H)** Colony formation assay in HCT116 cells pretreated with a PLK1 inhibitor (10 nM, 12 hours) followed by Wnt agonist 1 (2 nM, 12 hours), which rescued colony formation capacity after 14 days of culture. **(I-L)** CCK-8 assays showing rescued cell viability in CRC cell lines (RKO, HCT116, DLD1, and HT-29) treated with PLK1 inhibitors (BI2536, volasertib, onvansertib, and GSK461364; 10 nM, 12 hours) followed by the Wnt agonist 1 (2 nM, 12 hours) compared with that in the inhibitor-only groups. **(M)** Immunofluorescence analysis showing restored KI67 and p-H3(Ser10) fluorescence intensity in PLK1-overexpressing HCT116 cells treated with the Wnt agonist 1 (2 nM, 12 hours). **(N-O)** Characterization of a kinase-dead PLK1 mutant (PLK1-K82R) in PLK1-knockdown CRC cells. **(N)** Western blot analysis of PLK1 and p-PLK1 (T210) levels in PLK1-WT and PLK1-K82R-expressing cells treated with PLK1 inhibitors. **(O)** β-catenin protein levels in PLK1-WT and PLK1-K82R-expressing cells following PLK1 inhibition, demonstrating kinase activity-dependent regulation.

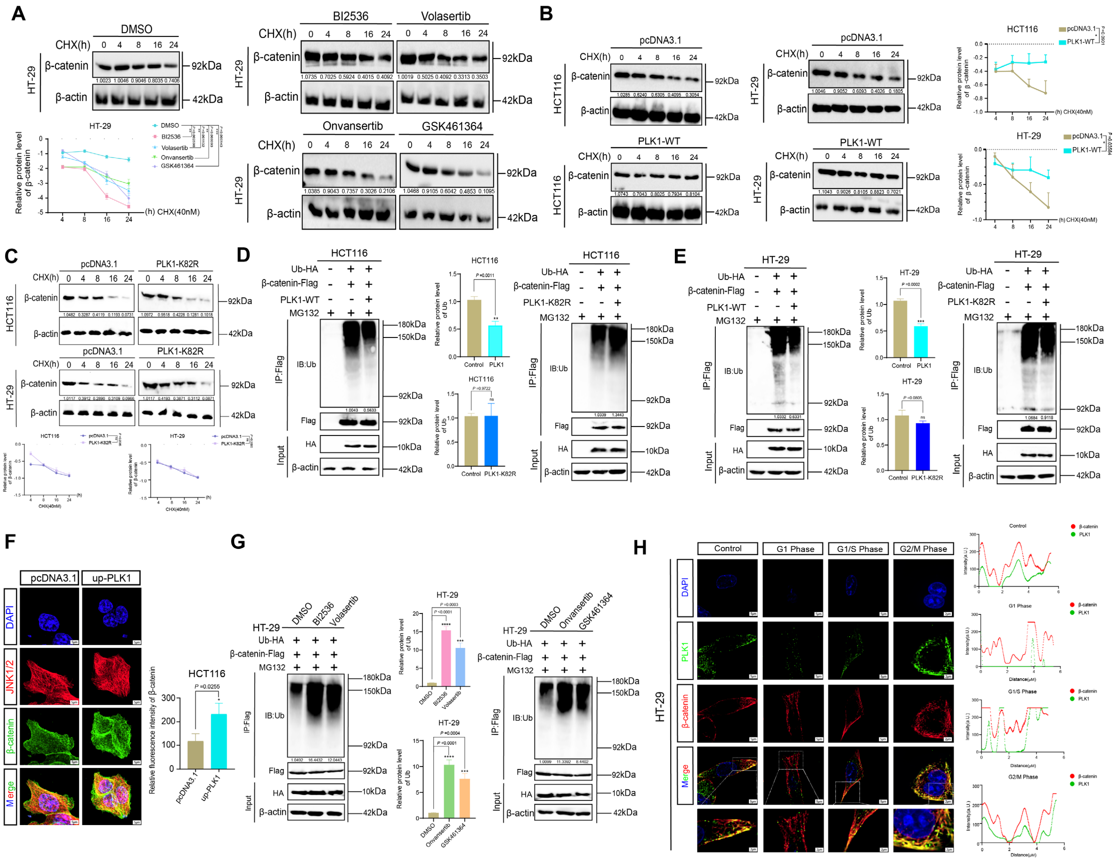

Figure S3. PLK1 stabilizes β-catenin by suppressing its ubiquitin-mediated degradation. (A) PLK1 inhibition (10 nM, 24 h) shortened the β-catenin half-life in HT-29 cells during the CHX (40 nM) chase assay. **(B)** Protein stability assay showing an extended β-catenin half-life in PLK1-WT-overexpressing HCT116/HT-29 cells treated with CHX (40 nM). **(C)** The kinase-dead PLK1-K82R mutant failed to extend the β-catenin half-life in HCT116/HT-29 cells, as shown by the CHX chase assay. **(D-E)** Ubiquitination assays demonstrated that PLK1-WT overexpression reduces β-catenin ubiquitination in HCT116 **(D)** and HT-29 **(E)** cells, whereas PLK1-K82R has no effect. The cells were treated with MG132 (10 nM, 48 h). **(F)** Confocal microscopy (SpinSR) revealed increased β-catenin fluorescence intensity in PLK1-overexpressing HCT116 cells. Scale bar: 5 µm. **(G)** PLK1 inhibition increases β-catenin ubiquitination in HT-29 cells coexpressing β-catenin-Flag and Ub-HA after MG132 treatment (10 nM, 48 h). **(H)** Confocal analysis of PLK1 and β-catenin colocalization across cell cycle phases (G1, G1/S, and G2/M) in synchronized HT-29 cells. Scale bar: 5 µm.

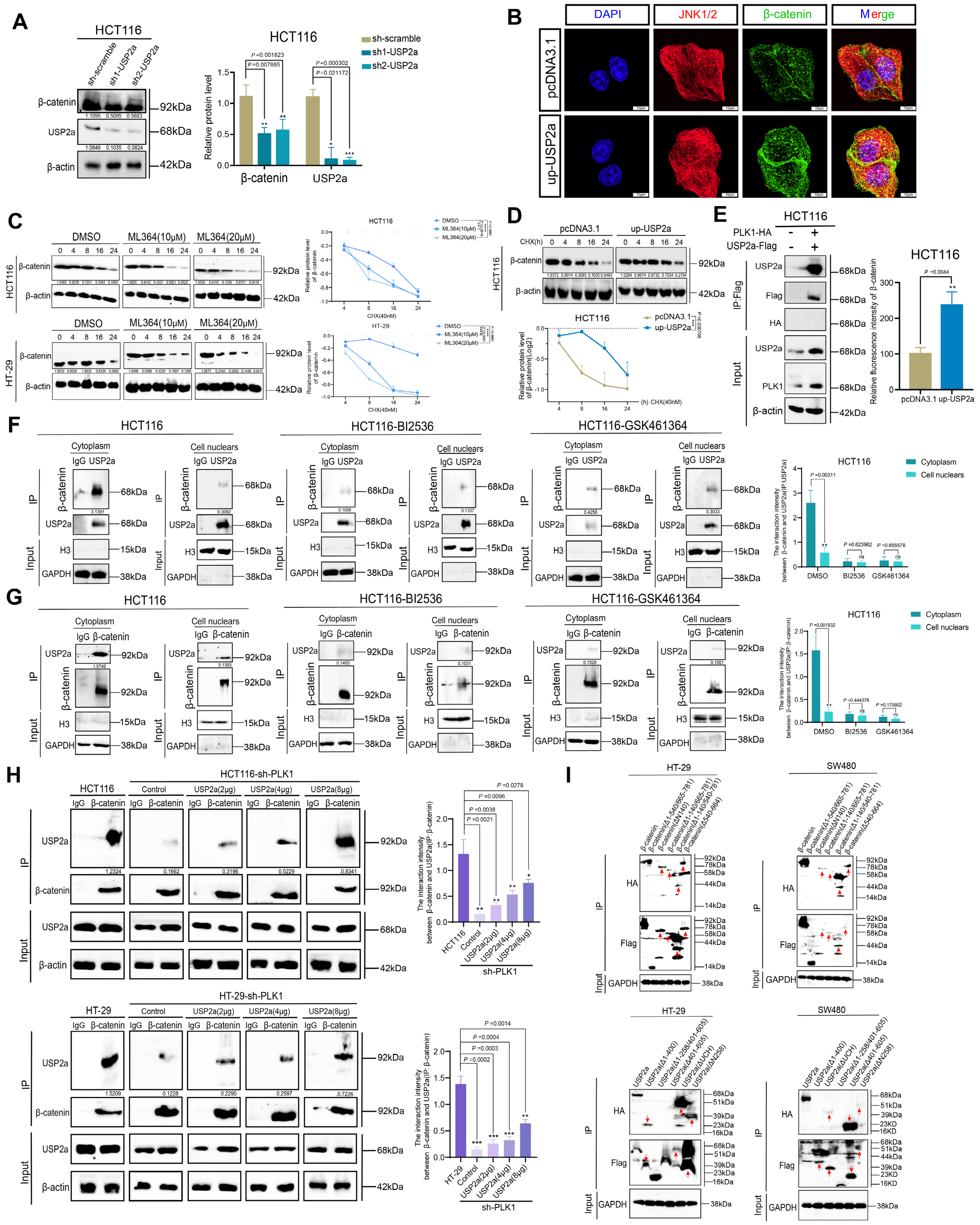

Figure S4. PLK1 attenuates β-catenin ubiquitination by upregulating USP2a. (A) USP2a knockdown reduces β-catenin protein levels in HCT116 cells (selected with 1 μM puromycin for 24 hours during 72-hour transfection). **(B)** Confocal microscopy (SpinSR) revealed increased β-catenin fluorescence intensity upon USP2a overexpression in HCT116 cells. Scale bar: 2 μm. **(C)** ML364 treatment (10 μM, 20 μM) shortened the β-catenin half-life in HCT116 cells in a concentration-dependent manner, as shown by the CHX (40 nM) chase assay. **(D)** USP2a overexpression extends the β-catenin half-life in HCT116 cells during the CHX (40 nM) chase assay. **(E)** Co-

immunoprecipitation revealed no direct interaction between USP2a and PLK1 in HCT116 cells co-expressing USP2a-Flag and PLK1-HA. **(F-G)** Subcellular fractionation followed by Co-IP revealed a predominant cytoplasmic interaction between USP2a and β-catenin in HCT116 cells. PLK1 inhibition with BI2536 or volasertib (10 nM, 16 h) reduces this interaction. **(H)** Dose-dependent Co-IP assay showing increasing USP2a–β-catenin binding with increasing USP2a concentrations (1–4 μg) in PLK1-knockdown HCT116 and HT-29 cells. The data were normalized and quantified. **(I)** Domain mapping in HT-29 and SW480 cells confirmed that β-catenin requires intact Armadillo repeats 1-10 and that USP2a requires the C-terminal domain for their interaction, as demonstrated by Co-IP with truncated mutants.

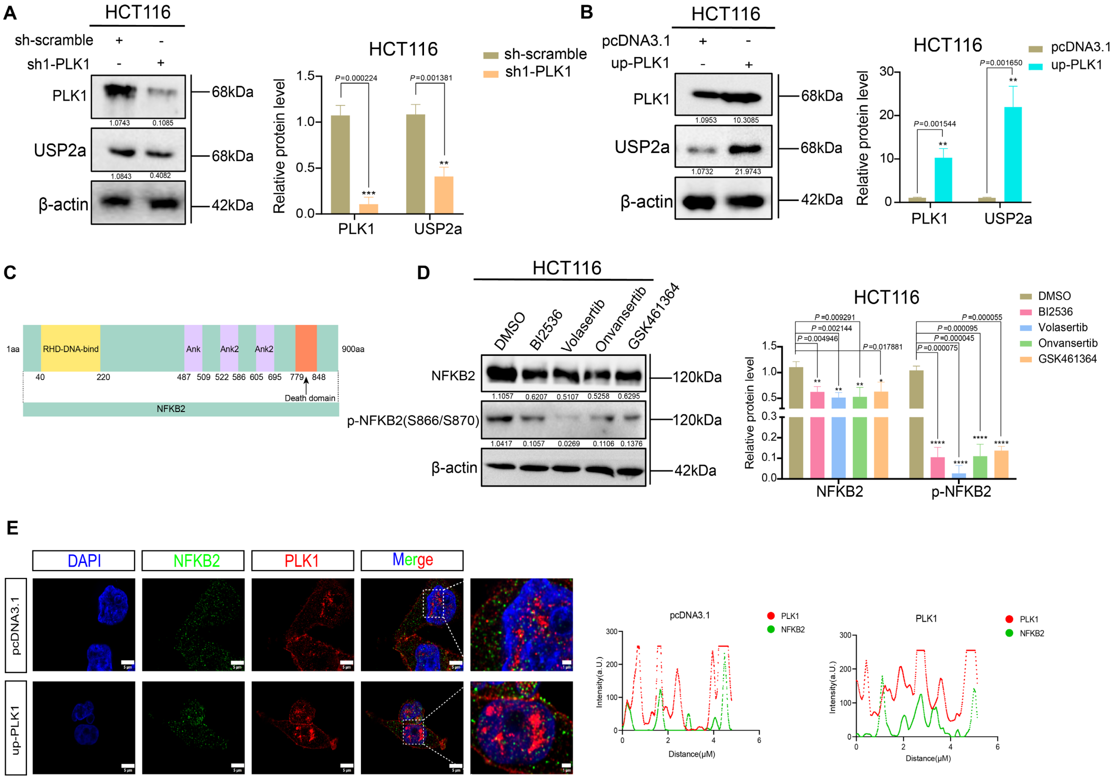

Figure S5. PLK1 activates NFKB2 to promote USP2a transcription. (A) PLK1 knockdown decreases USP2a protein levels in HCT116 cells (72-hour transfection with puromycin selection). **(B)** PLK1 overexpression increases USP2a protein levels in HCT116 cells (48 hours after transfection). **(C)** Schematic diagram of the NFKB2 protein structure. **(D)** PLK1 inhibition (10 nM, 24 hours) reduces NFKB2 phosphorylation levels in HCT116 cells. **(E)** Confocal microscopy (SpinSR) revealed enhanced PLK1-NFKB2 colocalization in PLK1-overexpressing HCT116 cells. The colocalization coefficients were quantified. Scale bar: 5 μm.

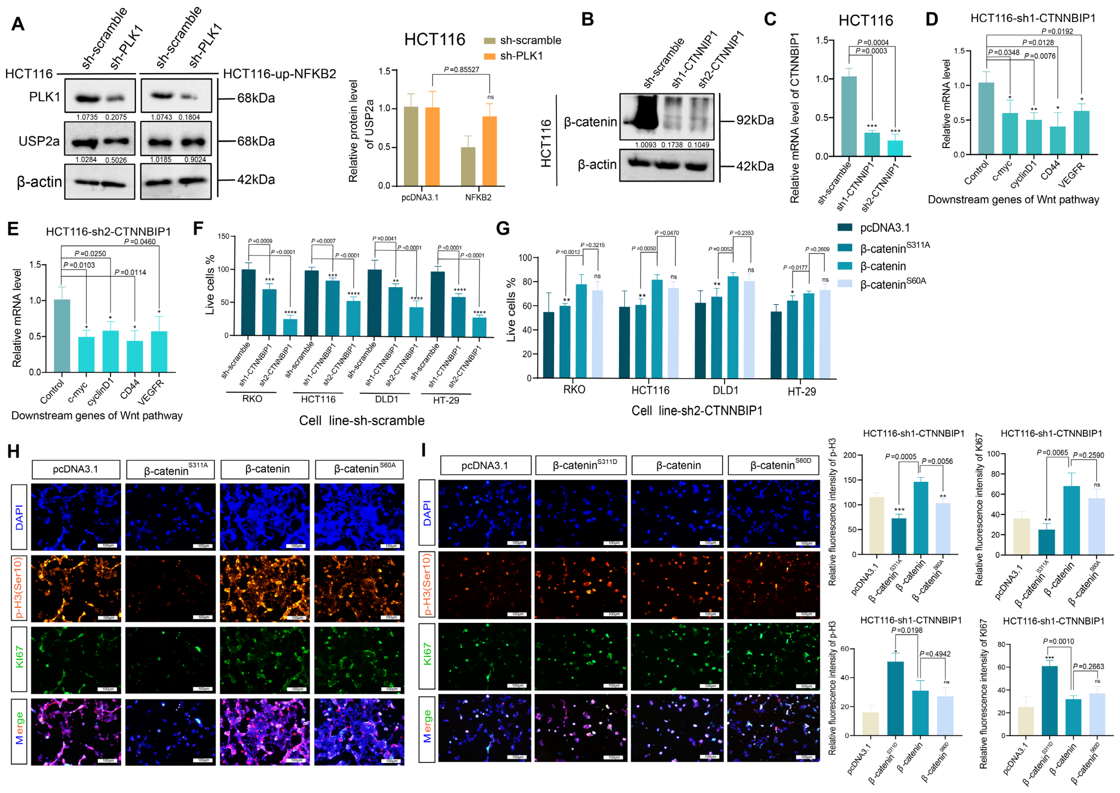

Figure S6. PLK1-mediated phosphorylation of β-catenin facilitates USP2a recruitment. (A) NFKB2 overexpression rescues USP2a protein levels in PLK1-knockdown HCT116 cells (72-hour transfection with puromycin selection). **(B)** Validation of β-catenin knockdown efficiency in stable HCT116 cell lines by Western blotting. **(C)** β-catenin mRNA levels in HCT116 β-catenin-knockdown cell lines. **(D-E)** qPCR analysis of Wnt/β-catenin downstream targets (c-Myc, Cyclin D1, CD44, and VEGFR) in two independent β-catenin-knockdown HCT116 cell lines: **(D)** HCT116-sh1-CTNNBIP1 and **(E)** HCT116-sh2-CTNNBIP1. **(F)** Cell proliferation was assessed by a CCK-8 assay in four CRC cell lines (RKO, HCT116, DLD1, and HT-29) following β-catenin overexpression. **(G)** CCK-8 assay showing growth differences in β-catenin-knockdown HCT116 cells reconstituted with wild-type β-catenin, β-catenin-S311A, or β-catenin-S60A mutants. **(H)** Immunofluorescence analysis of KI67 and p-H3(Ser10) intensity in β-catenin-knockdown HCT116 cells reconstituted with wild-type β-catenin, β-catenin-S311D, or β-catenin-S60D mutants. **(I)** Immunofluorescence analysis of proliferation markers in β-catenin-knockdown HCT116 cells reconstituted with wild-type β-catenin, β-catenin-S311A, or β-catenin-S60A mutants.

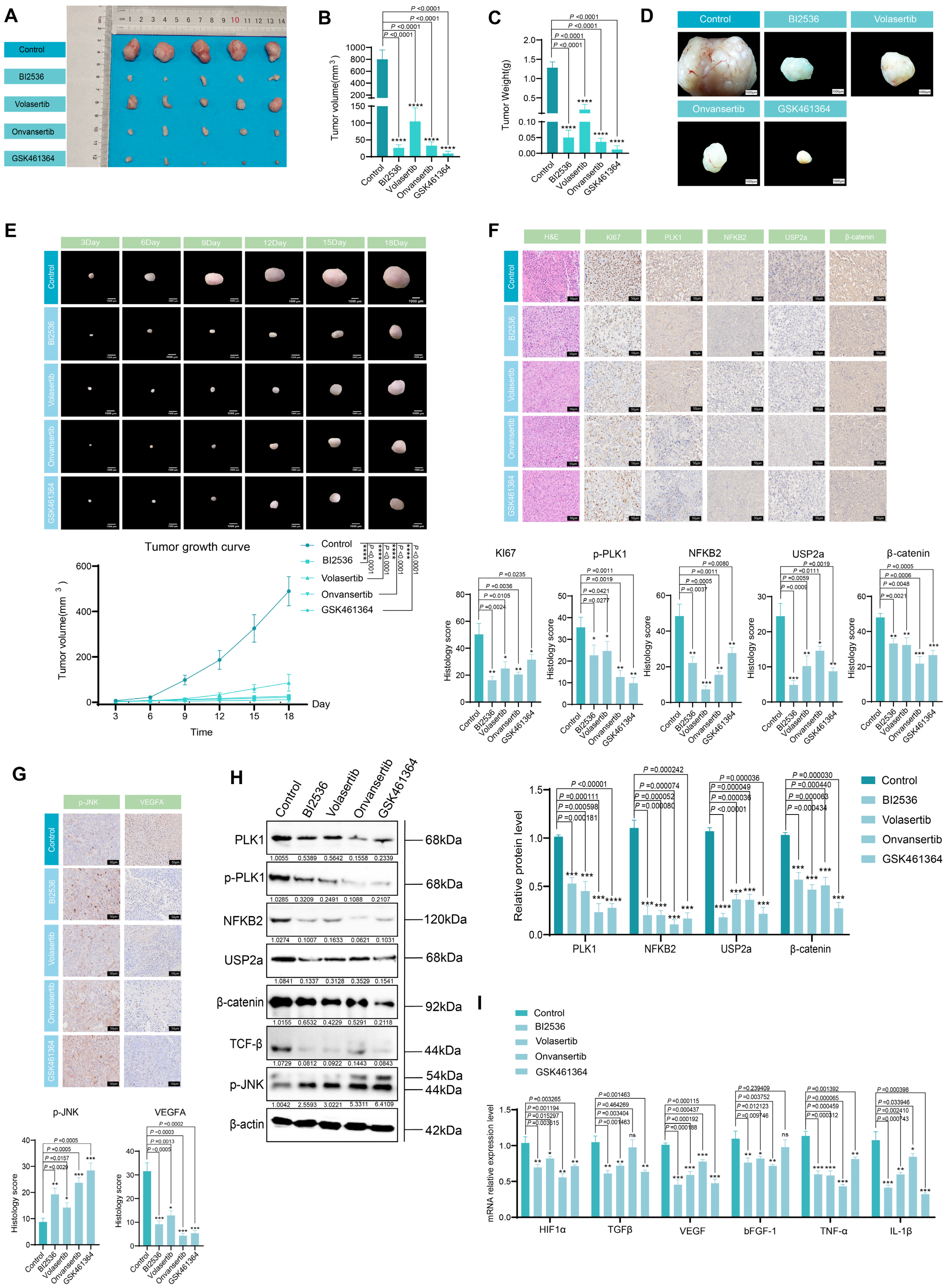

Figure S7. PLK1 inhibition suppresses tumor growth in CRC xenograft models. **(A)** *In vivo* therapeutic efficacy of PLK1 inhibitors. HCT116 xenograft-bearing mice were treated with BI2536 (50 mg/kg, i.v., every 3 days), volasertib (40 mg/kg, i.v., every 3 days), GSK461364 (10 mg/kg, i.v., daily), or onvansertib (10 mg/kg, p.o., daily) for 28 days. Representative images of dissected tumors are shown (n = 5 per group). **(B)** Tumor weights from different treatment groups. The data are presented as the means

± SEMs. **(C)** Final tumor volumes calculated via the formula $V = \pi/6 \times L \times W^2$ (mm^3). **(D)** Macroscopic examination of tumor vasculature from different treatment groups. **(E)** Tumor growth curves showing volume measurements taken every 3 days during the treatment period. The data represent the means ± SEMs. **(F)** Immunohistochemical staining of H&E, Ki67, PLK1, USP2a, β-catenin, and NFKB2 in xenograft tumor sections from different treatment groups. Scale bar: 20 μm. **(G)** Immunohistochemical analysis of p-JNK and VEGFA expression in xenograft tumors from different treatment groups. Scale bar: 20 μm. **(H)** Western blot analysis of PLK1, NFKB2, USP2a, β-catenin, p-JNK, and TGF-β protein expression in xenograft tumors from different treatment groups. **(I)** qPCR analysis of inflammatory factors (HIF1α, TGFβ, bFGF-1, TNF-α, and IL-1β) in xenograft tumors from different treatment groups. The data are presented as the means ± SEMs.

Supplemental Table 1 Primers for PCR and sequences for shRNAs

Primers for vector construction in this study

primers	Forward sequences（5’-3’）	Reverse sequences（5’-3’）
PLK1-Flag	CTAGCGTTTAAACTTAAGCTTGCCACCATGAGTGCTGCAGTGACTGC	TCAGAGATGAGTTTCTGCTCTGCGAATTCGGAGGCCTTGAGACGGTTG
PLK1-Myc	CTAGCGTTTAAACTTAAGCTTGCCACCATGAGTGCTGCAGTGACTGC	TTACAGATCCTCTTCAGAGATGAGTTTCT
USP2a-HA	CTTGGTACCGAGCTCGGATCCGCCACCATGCTTGTGCCCGGTT	AACATCGTATGGGTAGAATTCCATTCGGGAGGGCGGGCT
USP2a-Flag	GCCACCATGCTTGTGCCCGGTTCG	CATTCGGGAGGGCGGGCTGG
USP2a-V5	CTAGCGTTTAAACTTAAGCTTGCCACCATGCTTGTGCCC	GGTTTAAACGGGCCCTCTAGACATTCGGGAGGGCGGGCT
β-catenin-Flag	CTTGGTACCGAGCTCGGATCCGCCACCATGGCTACTCAAGCTGAT	AACATCGTATGGGTAGAATTCCAGGTCAGTATCAAACCAGGCC
β-catenin-HA	GCCACCATGGCTACTCAAGCTGATTTGATG	CAGGTCAGTATCAAACCAGGCC
β-catenin-Flag2	CTAGCGTTTAAACTTAAGCTTGCCACCATGGCTACTCAAGC	AACGGGCCCTCTAGACTCGAGCAGGTCAGTATCAAACCAGGCC
NFKB2-Myc1	ATGGAGAGTTGCTACAAC	TCAGTGCACCTGAGGCTG
NFKB2-Myc2	CTAGCGTTTAAACTTAAGCTTATGGAGAGTTGCTACAACCCAGG	GGTTTAAACGGGCCCTCTAGATCAGTGCACCTGAGGCTGGG
Catenin-S33A-Flag	GTCTTACCTGGACGCCGGAATCCAT	CGGGTCCAGGTAAGACTGTTGCTGC
Catenin-S60A-Flag	CGGGGTATCCACATCCTCTTCCTCA	CGGGGTATCCACATCCTCTTCCTCA
Catenin-S60D-Flag	GATGTGGATACCGACCAAGTCCTGT	CTGGGTATCCACATCCTCTTCCTCA
Catenin-S37A-Flag	TCTGGAATCCATGCCGGTGCCACTA	CGGATGGATTCCAGAGTCCAGGTAA
Catenin-T41A-Flag	TCTGGTGCCACTGCCACAGCTCCTT	CGGAGTGGCACCAGAATGGATTCCA
Catenin-S330A-Flag	AATATAATGAGGGCCTATACTTACG	CGGCCTCCGGCGGCAGAGCTCCAACACCAC
Catenin-S330D-Flag	AATATAATGAGGGACTATACTTACG	CTGCCTCATTATATTTACTAAAGCT
Catenin-S311A-Flag -	GGCAACCAAGAAGCCAAGCTCATCA	CGGTTCTTGGTTGCCATAAGCTAAA
β-catenin-1-141/540-781-Flag	CTTGGTACCGAGCTCCGCCACCATGGCTACTCAAGCTGAT	GTCATCCTTGTAATCGCAGGTCAGTATCAAACCAGGCC
β-catenin-1-540/665-781-Flag	CTTGGTACCGAGCTCCGCCACCATGGCTACTCAAGCTGAT	GTCATCCTTGTAATCGAATTCCAGGTCAGTATCAAACCAGGCC
β-catenin-1-664-Flag	CTTGGTACCGAGCTCCGCCACCATGGCTACTCAAGCTGAT	GTCATCCTTGTAATCGAATTCCATTCGGAACAAAACAGCAGC
β-catenin-141-664-Flag	CTTGGTACCGAGCTCCGCCACCATGTTGATTAACTATCAAG	GTCATCCTTGTAATCGAATTCCATTCGGAACAAAACAGCAGC
β-catenin-141-781-Flag	CTTGGTACCGAGCTCCGCCACCATGTTGATTAACTATCAAG	GTCATCCTTGTAATCGAATTCCAGGTCAGTATCAAACCAGGCC
PLK1-K82R-Flag	GGCCGTATTGTGCCTAAGTCTCTGCTGCTCAA	TTAGGCACAATACGGCCCGCGAACACCTCCTT
NFKB2-Flag	ATGGAGAGTTGCTACAACCCAGG	GGTTTAAACGGGCCCTCTAGATCAGTGCACCTGAGGCTGGG
NFKN2-S866A-Flag	AGCAGAGGTGAAGGAAGACGCC	TGGCTCCCGTACGCGGCGTCTTCCTT
NFKB2-S866D-Flag	AGCAGAGGTGAAGGAAGACGAC	TGGCTCCCGTACGCGTCGTCTTCCTT
USP2a-1-258-Flag	CTTGGTACCGAGCTCGGATCCGCCACCATGTCCCAGCTCT	GTCATCCTTGTAATCGAATTCCATGCCGTCTCTTCCCGG
USP2a-259-400-Flag	CTTGGTACCGAGCGCCACCATGAATTCTAAGAGTGC	GTCATCCTTGTAATCGAATTCCCACATCTGTCGGCCTTTCTC
USP2a-400-605-Flag	CTTGGTACCGAGCTCGGCCACCATGAGAAAATATCTAGAACG	GTCATCCTTGTAATCGAATTCCATTCGGGAGGGCGGGCT
USP2a-1-400-Flag	CTTGGTACCGAGCTCGGCCACCATGTCCCAGCTCT	GTCATCCTTGTAATCGAATTCCCACATCTGTCGGCCTTTCTC
USP2a-259-605-Flag	CTTGGTACCGAGCTCGGCCACCATGAATTCTAAGAGTGC	GTCATCCTTGTAATCGAATTCCATTCGGGAGGGCGGGCT
sh1-PLK1	CCGGGTCCATGGAAATATCCATTCTCGAGAATGGATATTTCCATGGACTTTTTG	AATTCAAAAAGTCCATGGAAATATCCATTCTCGAGAATGGATATTTCCATGGAC

sh2-PLK1	CCGGCGTGGAGCCCTCGATACATCTCGAGATGTATCGAGGGCTCCACGTTTTTG	AATTCAAAAACGTGGAGCCCTCGATACATCTCGAGATGTATCGAGGGCTCCACG
sh1-NFKB2	CCGGGAAGCGGAGGAAGGCCTTGCTCGAGCAAGGCCTTCCTCCGCTTCTTTTTG	AATTCAAAAAGAAGCGGAGGAAGGCCTTGCTCGAGCAAGGCCTTCCTCCGCTTC
Sh1-USP2	CCGGCTTCACCAAAGAGGATGTGCTCGAGCACATCCTCTTTGGTGAAGTTTTTG	AATTCAAAAACTTCACCAAAGAGGATGTGCTCGAGCACATCCTCTTTGGTGAAG
Sh2-USP2	CCGGGCCACTATACAGCCTACTGCTCGAGCAGTAGGCTGTATAGTGGCTTTTTG	AATTCAAAAAGCCACTATACAGCCTACTGCTCGAGCAGTAGGCTGTATAGTGGC
Sh1- CTNNBIP1	CCGGTGCTGAAACATGCAGTTGTCTCGAGACAACTGCATGTTTCAGCATTTTTG	AATTCAAAAATGCTGAAACATGCAGTTGTCTCGAGACAACTGCATGTTTCAGCA
Sh2- CTNNBIP1	CCGGCTGGCCATCTTTAAGTCTGCTCGAGCAGACTTAAAGATGGCCAGTTTTTG	AATTCAAAAACTGGCCATCTTTAAGTCTGCTCGAGCAGACTTAAAGATGGCCAG
USP2- Promote1	AACAGAGGTAATAGGCCGGGCGT	AGGACAGCGTCCGCGGCGCCCGAA
USP2-2000/100	CGGCGTAGAGGATCGAGATCTAACAGAGGTAATAGGCCGGGC	TTACCAACAGTACCGGAATTCAGGACAGCGTCCGCGGCG
USP2-1000/100	GTCAAAGACTGAGGGTGG	CGGCGTAGAGGATCGAGATCTGTCAAAGACTGAGGGTGGTG
USP2-500/100	ACACCAGAAGGAACAGAA	CGGCGTAGAGGATCGAGATCTACACCAGAAGGAACAGAAGG
USP2-250/100	TTATCCCGAGCTGAGCCT	CGGCGTAGAGGATCGAGATCTTTATCCCGAGCTGAGCCTCA
**Primers for RT-qPCR in this study**		
PLK1	ACGGCAGCGTGCAGATCAACTT	CCAGGAGACTCAGGCGGTATGT
FZD1	TGAGCCGACCAAGGTGTATG	CACTGACCAAATGCCAATCC
FZD3	ACGTGGATACAAGAACGC	CAGAGGGAATGCCAACTA
FZD4	GTGCCCTTACCTCACAAA	TAAGCCAGCATCATAGCC
FZD7	GACATCGCCTACAACCAGACC	CGCATACATGGAGCATAAGAAA
LGR4	TTCCCTAGTCATTCGTGG	GGTATGCTGCTTATCTTTG
NKD1	GCAAGATGCTGCGGGTAA	TCCTCAGTGGGCTTGGTCT
USP2	CCCCAATGATGTGGTGAG	CCAGAAGAAAGCGAAGGA
RBM3	GAGCAGGCACTGGAAGAC	CTGGGTTGGTGAAGGTGA
ZNF777	ACGAGTCCCTGGTTTCCAT	GCCCTCCTCAGAGTCTTCC
PES1	GACAATGGGTTCGTGGAG	GACAATGGGTTCGTGGAG
ARMC6	TGTGCAGGAGAACAAAGGC	CAGAACTCGTTGCGAATGG
BCL-9	GAAATGGTTCCACCTTCTATG	TGTTCCTGCTTCCTCCTCT
FOXO1	GTCCTACGCCGACCTCAT	TTGCTGTCACCCTTATCCTT
Myc	TCCTGTCCGTCCAAGCAG	ACGCACAAGAGTTCCGTAG
CD44	CAGATGGCATGAGGGATA	TGTGAGATTGGGTTGAAGA
NFKB2	GCCGAAAGACCTATCCCA	ATTGCTTGCCCACCAGAC
